# The Hox gene *Abdominal-B* uses Doublesex^F^ as a cofactor to promote neuroblast apoptosis in the *Drosophila* central nervous system

**DOI:** 10.1242/dev.175158

**Published:** 2019-08-22

**Authors:** Neha Ghosh, Asif Bakshi, Risha Khandelwal, Sriivatsan Govinda Rajan, Rohit Joshi

**Affiliations:** 1 Laboratory of Drosophila Neural Development, Centre for DNA Fingerprinting and Diagnostics (CDFD), Inner Ring Road, Uppal, Hyderabad 500039, India; 2 Graduate Studies, Manipal Academy of Higher Education, Manipal 576104, India; 3 Department of Biological Sciences, University of Illinois at Chicago, Chicago, IL 60607, USA

**Keywords:** Abdominal-B, Doublesex, *Drosophila*, Neuroblast, Apoptosis, Sexual dimorphism

## Abstract

Highly conserved DM domain-containing transcription factors (Doublesex/MAB-3/DMRT1) are responsible for generating sexually dimorphic features. In the *Drosophila* central nervous system, a set of Doublesex (Dsx)-expressing neuroblasts undergo apoptosis in females whereas their male counterparts proliferate and give rise to serotonergic neurons crucial for adult mating behaviour. Our study demonstrates that the female-specific isoform of Dsx collaborates with Hox gene *Abdominal-B* (*Abd-B*) to bring about this apoptosis. Biochemical results suggest that proteins AbdB and Dsx interact through their highly conserved homeodomain and DM domain, respectively. This interaction is translated into a cooperative binding of the two proteins on the apoptotic enhancer in the case of females but not in the case of males, resulting in female-specific activation of apoptotic genes. The capacity of AbdB to use the sex-specific isoform of Dsx as a cofactor underlines the possibility that these two classes of protein are capable of cooperating in selection and regulation of target genes in a tissue- and sex-specific manner. We propose that this interaction could be a common theme in generating sexual dimorphism in different tissues across different species.

## Abstract (precis)

**Highlighted Article:** *Drosophila* Doublesex^F^ collaborates with *Abdominal-B* to generate a sexually dimorphic central nervous system.

## INTRODUCTION

Sexual reproduction is central to animal existence and propagation. Irrespective of the different upstream molecular events, generation of sexually dimorphic traits converge down to a highly conserved DM domain containing non-classical zinc-finger proteins such as Doublesex (Dsx) in *Drosophila*, Male abnormal 3 (MAB-3) in *Caenorhabditis* *elegans* and Doublesex/Male-abnormal-3 Related Transcription factor 1 (Dmrt1) in vertebrates (Matson and Zarkower, 2012). The DM domain is an intertwined zinc finger-containing DNA binding module (Erdman and Burtis, 1993; Zhu et al., 2000) associated with sexual development and reproduction across different species (Baker and Ridge, 1980; Burtis and Baker, 1989; Shen and Hodgkin, 1988; Raymond et al., 2000; Chong et al., 2013; Matson and Zarkower, 2012; Raymond et al., 1999; Ross et al., 2005).

Apoptosis also plays an important role in generating sexual dimorphism in neural and non-neural tissues of *Drosophila* (DeFalco et al., 2003; Billeter et al., 2006b; Billeter et al., 2006a; Wang et al., 2011; Birkholz et al., 2013a; Kimura et al., 2005; Ren et al., 2016; Truman et al., 1993), *C. elegans* (Peden et al., 2007; Conradt and Horvitz, 1999) and vertebrates (Davis et al., 1996; Price et al., 1977; Roberts et al., 1999). *Drosophila* is a useful system for studying sexual dimorphism, wherein Dsx is an executive transcription factor (TF) of the sex determination hierarchy. Male and female isoforms of Dsx have a common region containing DNA-binding and dimerization domains followed by a sex-specific C-terminal region responsible for generating sex-specific features (Burtis and Baker, 1989; Erdman and Burtis, 1993). The Hox gene *Abdominal-B* (*Abd-B*) and its homologues are also known for their role in generating sexual dimorphism across different species (Kalis et al., 2010; Satokata et al., 1995; Dolle et al., 1991; DeFalco et al., 2004; Wang and Yoder, 2012; Wang et al., 2011; Jeong et al., 2006; Williams et al., 2008; Kopp et al., 2000; Tanaka et al., 2011; Foronda et al., 2012). Proteins Dsx and AbdB are known to function together in cuticle pigmentation as well as in growth and differentiation of *Drosophila* genital discs (Sanchez et al., 2001; Kopp et al., 2000; Williams et al., 2008). However, the precise details of Dsx and AbdB interaction remains unclear. Similarly, although the role of Dsx in generating sexual dimorphism in the central nervous system (CNS) has been well investigated (Lee et al., 2002; Baker and Ridge, 1980; Taylor and Truman, 1992; Villella and Hall, 1996; Billeter et al., 2006b; Waterbury et al., 1999; Zhou et al., 2014; Rideout et al., 2010), its possible interaction with a spatial determinant such as AbdB has not been tested.

In *Drosophila*, neural stem cells (neuroblasts, NBs) in abdominal segments (A3-A7) of the larval ventral nerve cord (VNC) undergo Hox gene *abdominal-A* (*abd-A*)-mediated apoptosis in the late larval stage (Bello et al., 2003). This apoptosis is mediated by the RHG family genes *grim* and *reaper*, activated through an enhancer lying within a 22 kb genomic region called the neuroblast regulatory region (NBRR) (Arya et al., 2015; Tan et al., 2011). It has been subsequently shown that AbdA, along with another TF Grainyhead (Grh), transcriptionally activates apoptotic genes through a 1 kb enhancer located within the NBRR (Khandelwal et al., 2017).

**Fig. 1.**
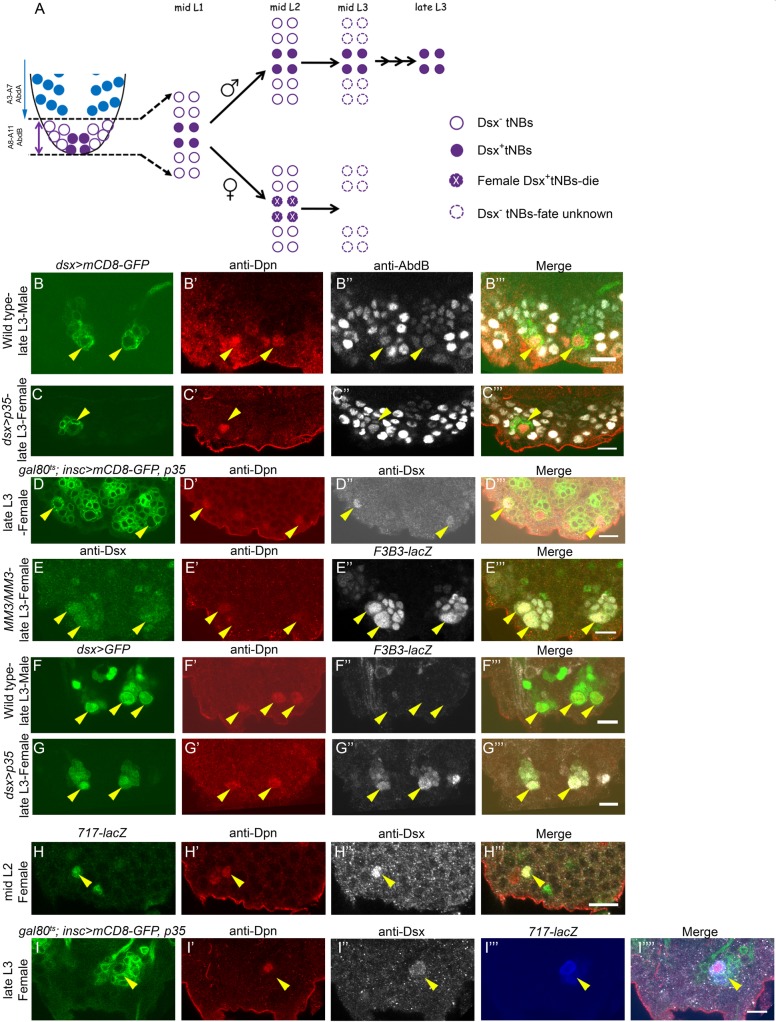
**Sexually dimorphic expression of abdominal apoptotic enhancer in Dsx^+^tNBs.** (A) Schematic of terminal segments (A8-A11) of larval VNC. (B,C) Dsx^+^tNBs are marked by *dsx-GAL4* and express AbdB in males (*n*=10) (B″) and females (*n*=11) (C″). In females, cell death was blocked by p35. (D) Blocking cell death from mid-L2 rescues Dsx^+^tNBs in female VNCs (see Fig. S2B for TS protocol, *n*=10)*.* (E) *F3B3-lacZ* expresses in Dsx^+^tNBs of *MM3* mutant female VNCs (*n*=11) until late L3. (F,G) *F3B3-lacZ* shows sex-specific expression in Dsx^+^tNBs of female VNCs (*n*=10) (G″) but not in male VNCs (*n*=11) (F″). (H) *717-lacZ* is expressed in mid-L2, prior to apoptosis in Dsx^+^tNBs of female VNCs (*n*=12). (I) *717-lacZ* sustains its expression in Dsx^+^tNBs in female VNCs until late L3 when apoptosis is blocked (Fig. S2A for TS protocol, *n*=8). *n* number of VNCs analysed. Yellow arrowheads indicate Dsx^+^tNBs. Scale bars: 10 µm. All images are single confocal sections.

In terminal segments (A8-A10) of the embryonic VNC, which expresses *Abd-B*, a subpopulation of 62 NBs (Birkholz et al., 2013a) undergo apoptosis while remaining cells enter quiescence (Monedero Cobeta et al., 2017). Only 12 of these 62 NBs exit quiescence in early larval stages and are referred to as terminal NBs (tNBs) (Taylor and Truman, 1992; Truman and Bate, 1988). Four of these 12 tNBs express the *doublesex* (*dsx*) gene and are referred to hereafter as Dsx^+^tNBs. In females, these cells undergo apoptosis mediated by Dsx^F^, whereas Dsx^M^ mediates their continued proliferation in males until the late third instar larval stage (L3) (Fig. 1A) (Taylor and Truman, 1992; Truman and Bate, 1988; Birkholz et al., 2013a), resulting in additional serotonergic neurons that are crucial for male mating behaviour in adults (Billeter et al., 2006a,b). Interestingly, although Dsx-mediated sex-specific neuronal apoptosis occurs across different regions of the developing CNS (Billeter et al., 2006b; Kimura et al., 2008; Sanders and Arbeitman, 2008), Dsx-mediated larval NB apoptosis has so far been reported specifically in females and only in the AbdB-expressing region of the CNS (Birkholz et al., 2013a; Taylor and Truman, 1992; Truman and Bate, 1988). This offers a unique opportunity to understand if (and how) spatial patterning genes could collaborate with the sex determination hierarchy to pattern the CNS.

In the current body of work, we investigate the molecular mechanism of female-specific apoptosis of Dsx^+^tNBs in the larval CNS. We found that Hox gene *Abd-B* uses Dsx^F^ as a cofactor to cause sex-specific transcriptional activation of the RHG family of apoptotic genes, to generate sexual dimorphism in the structure of the developing CNS. Biochemical results suggest that AbdB and Dsx are capable of physically interacting with each other through their highly conserved homeodomain (HD) and DM domain, respectively. This interaction translates into cooperative binding of the two proteins on the DNA, which *in vivo* translates into sex-specific activation of the apoptotic enhancer in females. Congruent to this, mutagenesis of cooperative binding motifs is sufficient to abrogate female-specific apoptotic enhancer activity *in vivo*.

Collectively, our insights suggest that spatial determinants such as AbdB can utilize the sex-specific isoform of Dsx as a cofactor to select and activate the respective target genes in a tissue- and sex-specific manner. Considering the wide-ranging role of the *dsx* gene, we suggest that its capacity to collaborate with region-specific factors such as the Hox genes (in this case, AbdB) or other HD proteins could be a common theme for generating sexual dimorphism during development.

## RESULTS

### Sexually dimorphic expression of abdominal apoptotic enhancer in Dsx^+^tNBs

Dsx-expressing embryonic NBs express AbdB (Birkholz et al., 2013a), therefore we started out by testing whether Dsx and AbdB are also co-expressed in larval NBs. We found that NBs (marked by Deadpan, Dpn) in all of the four *dsx-GAL4*-marked tNB lineages (*dsx-GAL4>mCD8-GFP*) expressed Hox gene *Abd-B* in both males (Fig. 1B-B‴) and females (Fig. 1C-C‴). This was in contrast to abdominal NBs, which are Hox^−^ and die on becoming Hox^+^ (or AbdA^+^) (Bello et al., 2003). To establish the precise time of apoptosis of Dsx^+^tNBs in female VNC, we expressed cell death blocker p35 in larval stages using a temporally inducible GAL4 system [*tub-GAL80^ts^; insc-GAL4* used in temperature shift (TS) experiments]. We recovered all four Dsx^+^tNBs (4.0±0.89, *n*=12, *N*=3 where *n* is the number of VNCs analysed and *N* the number of experiments) for an early first instar larva stage (L1) TS (for TS experimental protocol, see Fig. S2A), an average of three of these cells (2.8±0.92, *n*=10, *N*=3) for mid-L2 TS (Fig. S2B; Fig. 1D-D‴) and none in early L3 TS (Fig. S2C), suggesting that these cells undergo apoptosis in approximately mid- to late L2.

Larval abdominal NBs apoptosis in A3-A7 segments requires *grim* and *reaper* expression, through an apoptotic enhancer lying within a 22 kb NBRR. A 54 kb genomic deletion (*MM3*) deletes the NBRR (Tan et al., 2011), whereas a smaller deletion called *M22* deletes a 14.5 kb region within the NBRR. We found that female larval VNCs of genotype *M22/MM3* show a block of Dsx^+^tNBs apoptosis (Fig. S1A-A″), suggesting that the apoptotic enhancer crucial for their death also lies within the 14.5 kb region uncovered by *M22* deletion. A 1 kb region (referred to as *F3B3*) within *M22* was identified as the abdominal NB apoptotic enhancer (Khandelwal et al., 2017). We tested and found that the 1 kb region (*F3B3-lacZ*) and its 717 bp subfragment (*717-lacZ*) were also expressed in Dsx^+^tNBs at the mid-L2 stage in female VNCs prior to cell death; thereby establishing precise temporal expression of the enhancer (1.5±0.52 Dsx^+^lacZ^+^tNBs; *n*=12 VNCs, *N*=3) (Fig. 1H-H‴). A hallmark of an apoptotic enhancer is its capacity to sustain expression until the target cells undergo apoptosis. Congruent to this, we observed that *F3B3-lacZ* and *717-lacZ* were expressed and sustained until late L3 in all four Dsx^+^tNBs when cell death was blocked in female VNCs either by *MM3* deletion (Fig. 1E-E‴) or by p35 expression (Fig. 1I-I″″) (Fig. S2A for TS protocol; 4.0±0.5 Dsx^+^lacZ^+^tNBs, *n*=8 VNCs, *N*=4).

Dsx^+^tNB apoptosis is female-specific (Birkholz et al., 2013a) and, as expected, we observed that *F3B3-lacZ* and *717-lacZ* were specifically expressed in Dsx^+^tNBs of female VNCs (Fig. 1G″ and Fig. 1I‴), but not in male VNCs (Fig. 1F″ and Fig. S1B).

Collectively, these results suggested that AbdB and Dsx express in larval tNBs and that the abdominal apoptotic enhancer is activated in a sexually dimorphic fashion to cause death of Dsx^+^tNBs from the mid-L2 stage in females.

### Dsx^+^tNB apoptosis in females requires AbdB but not Exd, Hth, Grh and Notch

**Fig. 2.**
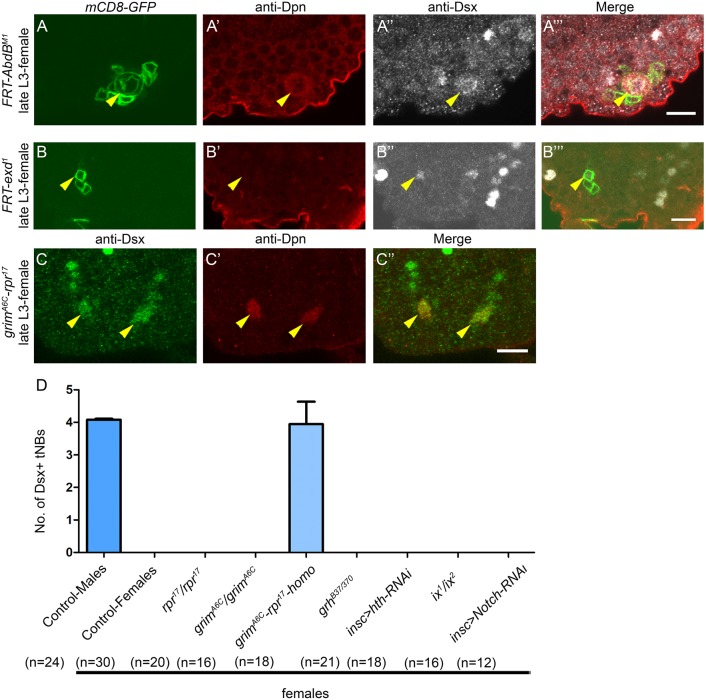
**Dsx^+^tNB apoptosis in females requires AbdB and occurs through RHG genes *grim* and *reaper*.** (A) Dsx^+^tNB mutants for *AbdB^M1^* do not undergo apoptosis in female VNCs (*n*′=9). (B) *exd^1^* mutant Dsx^+^tNBs die normally (*n*′=12). GFP-marked *exd^1^* mutant lineage expressing Dsx protein and lacking the NB is shown. (C) *grim^A6C^-rpr^17^* double-mutant homozygous female VNCs (*n*=18) exhibit a block of Dsx^+^tNB apoptosis. (D) Quantification of the Dsx^+^tNBs across various genotypes in the female VNC. *n* number of VNCs, *n*′ number of MARCM clones analysed. Yellow arrowheads indicate Dsx^+^tNBs. Scale bars: 10 µm. All images are single confocal sections. Graphs show mean±s.d.

Because Dsx-mediated NB cell death is reported only in the AbdB-expressing region of the female larval CNS, we tested the role of AbdB in sex-specific apoptosis. To this end, MARCM clones were made for the loss-of-function *AbdB^M1^* allele. We could successfully recover a Dsx-expressing NB clone mutant for the *AbdB^M1^* allele in the terminal region of the female larval CNS (Fig. 2A-A‴) (9 MARCM clones analysed from 16 VNCs) showing that AbdB is crucial for apoptosis. Next, we tested known Hox cofactors Extradenticle (Exd) and Homothorax (Hth) for their expression (Fig. S4) and role in this apoptosis. In the case of *exd^1^* mutant MARCM clones analysed in late L3, we could recover remnants of GFP-marked lineages that stained for Dsx, but none of these lineages contained any surviving NBs (12 lineages from 26 VNCs) (Fig. 2B-B‴). However, we recovered abdominal mutant clones with surviving NBs, showing that the mutant line was working (Fig. S5). This indicated that Dsx^+^tNBs divided before undergoing apoptosis and that Exd does not play a role in this apoptosis. Next, we tested whether Hth could substitute for Exd as a Hox cofactor. This was done by making *hth^P2^* MARCM clones. Here again, we did not recover any Dsx^+^ lineages in female VNCs. RNA interference (RNAi)-mediated knockdown of Hth protein from early L1 (Fig. S2A for TS protocol) did not yield any Dsx^+^ lineages (*n*=18 VNCs, *N*=3) in female VNCs, although there was a potent knockdown of Hth protein in larval thoracic NBs (Khandelwal et al., 2017).

Because Grh and Notch have been shown to work with AbdA in abdominal NBs apoptosis (Khandelwal et al., 2017), we assessed their role in Dsx^+^tNB apoptosis in females. Both Grh and Notch were expressed in Dsx^+^tNBs in mid-L2 (Fig. S4). Neither a CNS-specific null allelic combination of *grh* (*grh^370/B37^*) (Fig. 2D; *n*=21, *N*=2) nor knockdown of *Notch* using RNAi (from early L1; Fig. S2A for TS protocol) resulted in any ectopic Dsx^+^tNB apoptosis in female VNCs in late L3 (Fig. 2D; *n*=12, *N*=3). However, we did recover abdominal NBs in both the cases (Fig. S5).

Subsequently, homozygous deletions for RHG genes *grim*, *reaper* and *grim-reaper* double mutants were tested. We recovered four Dsx^+^tNBs only for the homozygous double deletion (*grim-reaper*) (Fig. 2C-C″ and Fig. 2D; 3.94±0.64, *n*=18 VNCs, *N*=3), suggesting that both *grim* and *reaper* are required for Dsx^+^tNB apoptosis in females (as in the case of abdominal NB cell death).

Collectively, these results show that Dsx^+^tNBs seem to rely only on Hox (AbdB) and Dsx^F^ for sex-specific activation of apoptotic genes *grim* and *reaper* in female VNCs, unlike apoptosis in abdominal NBs that depends on Hox (AbdA), Exd, Grh and Notch.

### AbdB regulates Dsx in tNBs

**Fig. 3.**
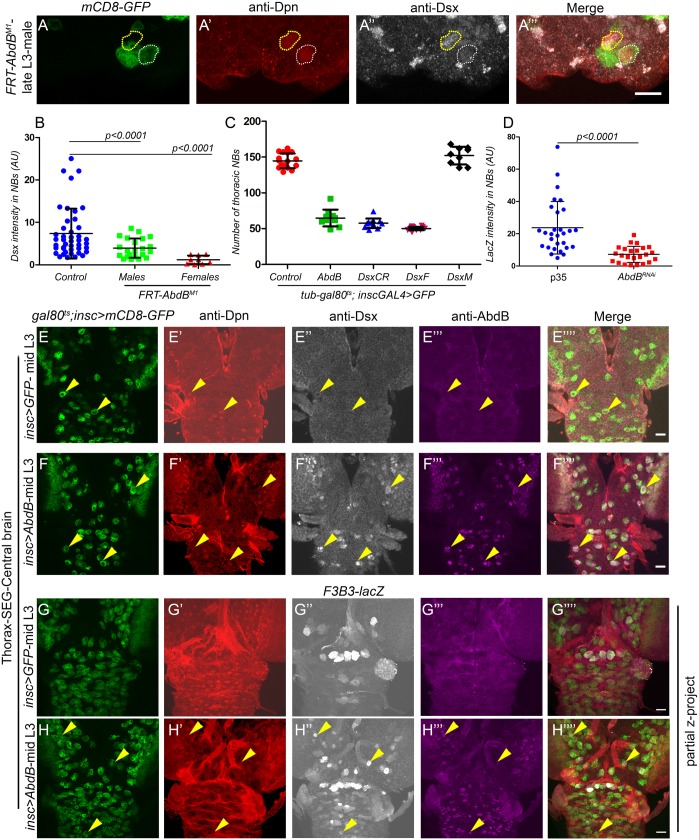
**AbdB regulates Dsx in tNBs.** (A) Dsx^+^tNB mutant for *AbdB^M1^* in male VNCs (*n*′=22) (white dotted line) show a decrease in levels of Dsx compared with adjacent wild-type control cells (yellow dotted line). (B) Graph comparing Dsx intensity in control NBs (*n*′=43) (in male VNCs) versus the NB mutant for *AbdB^M1^* in male (*n*′=22) and female VNCs (*n*′=9). (C) Graph comparing the total number of thoracic NBs found in control VNC (*n*=17) versus AbdB (*n*=10), Dsx^CR^ (*n*=12), Dsx^F^ (*n*=14) and Dsx^M^ (*n*=9) overexpressing male VNCs at late L3 (Fig. S2E for TS protocol for AbdB and Fig. S2B for others). (D) Graph comparing *F3B3-lacZ* intensity in Dsx^+^tNBs in female VNCs as control (blocked for apoptosis using p35, *n*=9) versus *AbdB* knockdown (*n*=9) (Fig. S2A for TS protocol). (E-H) Ectopic expression of AbdB in anterior region of CNS induces Dsx (*n*=12) (F″) and *F3B3-lacZ* (*n*=8) (H″) in NBs compared with wild-type controls (*n*=11 for E″, *n*=10 for G″) (Fig. S2E for TS protocol). Both male and female VNCs were analysed. Representative data and images shown here are from males, except in B where both male and female data are shown and in D where only female data is shown. *n* number of VNCs, *n*′ number of MARCM clones analysed. Yellow arrowheads indicate NBs. Scale bars: 10 µm for A; 20 µm for E-H. All images are single confocal sections except panels G and H. Graphs show mean±s.d. Significance (*P-*value) is from two-tailed Student's unpaired *t*-test.

AbdB is known to regulate Dsx expression in non-neural tissues (Kopp et al., 2000; Wang et al., 2011; Wang and Yoder, 2012; Tanaka et al., 2011; Foronda et al., 2012); therefore, we tested regulation of Dsx expression in Dsx^+^tNBs by checking *AbdB^M1^* MARCM clones in both females and males. We observed a decrease in Dsx staining in *AbdB^M1^* mutant NBs (marked by mCD8-GFP) in both female (Fig. 2A″) and male VNCs (Fig. 3A″). Quantitative comparison showed that mutant NBs of both males (3.97±2.271, 22 MARCM clones analysed in 21 VNCs, *N*=4) and females (1.18±1.01, 9 clones in 16 VNCs, *N*=4) demonstrated a significant decrease in Dsx levels compared with control NBs (7.35±5.87, 20 clones in 43 male VNCs, *N*=4) (Fig. 3B). Next, ectopic expression of AbdB was tested for its capacity to induce Dsx in NBs of the anterior CNS (thorax, subesophageal ganglia and central brain region), where Dsx is not normally expressed (in NBs). We observed that induction of AbdB (Fig. S2E for TS protocol) in the anterior regions of the CNS (Fig. 3F‴) ectopically induced Dsx expression in NBs in both males and females compared with controls (Fig. 3E″ versus 3F″; *n*=12 VNCs, *N*=2).

Ectopic expression of AbdB in the anterior region of the CNS is known to cause apoptosis of thoracic NBs (Bello et al., 2003). Congruent to this we also found that AbdB could induce apoptotic enhancer *F3B3-lacZ* in anterior regions of the CNS both in males (Fig. 3H″, *n*=8 VNCs, *N*=2) and females, as scored by an increase in intensity of *lacZ* and number of *lacZ*-expressing cells (Fig. 3G″ versus 3H″). The expression of *F3B3-lacZ* seen in control CNS (Fig. 3G″) was background leaky expression of the *enhancer-lacZ*. Comparison of the number of surviving GFP-marked thoracic NB lineages for control (144.6±2.5 NBs, *n*=17 VNCs, *N*=3) versus AbdB overexpression in males (64.80±3.7 NBs, *n*=10 VNCs, *N*=3; Fig. 3C) and females (69.8±8.8, *n*=12 VNC, *N*=3), showed a marked decrease in the latter two cases. Comparison of *F3B3-lacZ* expression in Dsx^+^tNBs for AbdB knockdown by RNAi versus p35-expressing controls was carried out for female VNCs in late L3 (Fig. S2A for TS protocol). Analysis showed that AbdB-RNAi could consistently downregulate *F3B3-lacZ* in NBs (7.19±5.0; measured from 24 NBs, *n*=9 VNCs, *N*=3) compared with p35-expressing controls (23.6±16.2; measured from 28 NBs, *n*=9 VNCs, *N*=3; Fig. 3D). A similar experiment could not be done for Dsx because none of the RNAi lines tested were potent enough to significantly knock down Dsx expression in NBs.

These results show that AbdB regulates Dsx expression in tNBs of both the male and female CNS. The ectopic induction and knockdown experiments suggest that AbdB can transcriptionally regulate the apoptotic enhancer in Dsx^+^tNBs of female VNCs.

### Dsx common region is enough to cause NB apoptosis

**Fig. 4.**
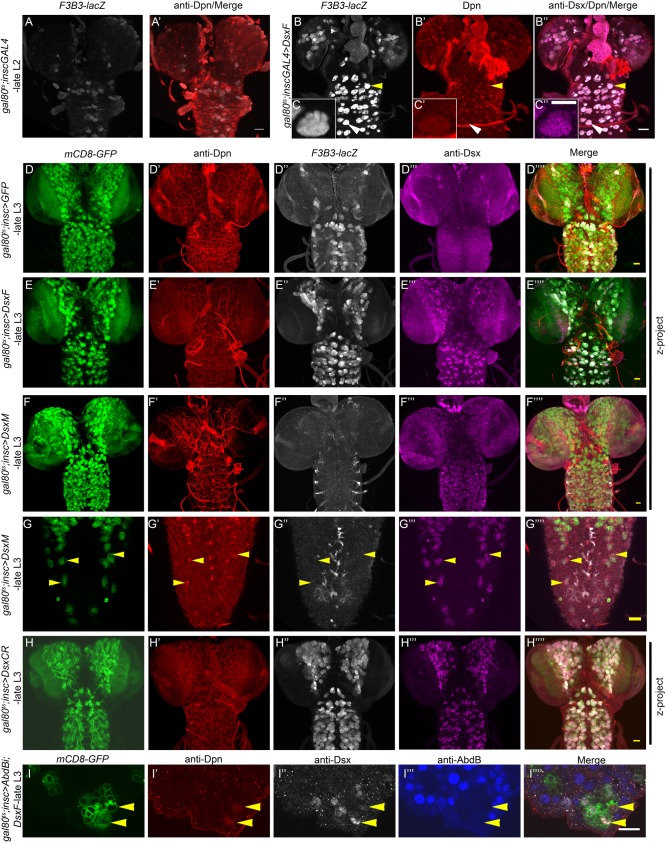
**Role of sex-specific region of Dsx in apoptosis.** (A,B) Compared with controls (*n*=10) (A,A′), induction of Dsx^F^ in mid-L2 (B-B″) activates *F3B3-lacZ* in NBs of the anterior CNS in late L2 (*n*=14) (Fig. S2F for TS protocol). (C) Induction of *F3B3-lacZ* in thoracic NBs in response to ectopic expression of Dsx^F^. (D,E,H) Compared with controls, misexpression of both Dsx^F^ (*n*=17) and Dsx^CR^ (*n*=12) cause induction of *F3B3-lacZ* (D″ vs E″; D″ vs H″) as well as NB apoptosis in the anterior CNS at late L3 (D vs E; D vs H) (Fig. S2B for TS protocol). (F) Ectopic expression of Dsx^M^ (*n*=11) in the anterior CNS (F‴) represses the leaky expression of *F3B3-lacZ* in thoracic segments (F″) and does not cause apoptosis of thoracic NBs (F). (G) Dsx^M^ also represses *F3B3-lacZ* (*n*=12) (G″) and blocks apoptosis of abdominal NBs (G). (I) Simultaneous overexpression of Dsx^F^ and knockdown of AbdB in the female VNCs (*n*=8) blocks apoptosis of tNBs (Fig. S2A for TS protocol), suggesting that both AbdB and Dsx^F^ are required for NB apoptosis. Both male and female VNCs were analysed. Representative data and images are shown here are from males, except in F,G,I where only female data is shown. *n* number of VNCs analysed. Yellow arrowheads indicate NBs. Scale bars: 20 µm for A,B,D-H; 10 µm for C,I. All images are single confocal sections except panels D,E,F,H.

**Fig. 5.**
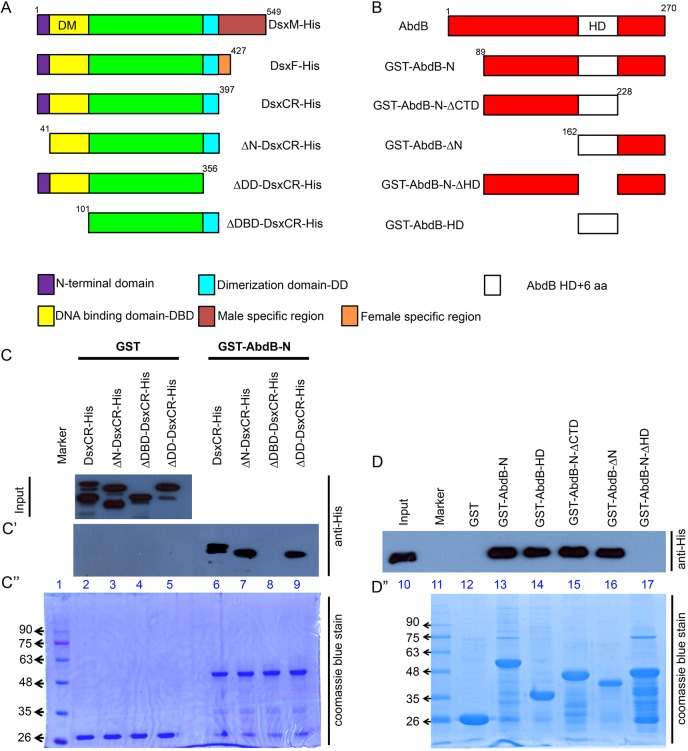
**AbdB and Dsx interact physically *in vitro*.** (A,B) Schematic of full-length and domain-deleted proteins used in the case of Dsx (A) and AbdB-r (B). (C,D) Western blot of the GST pulldown for bacterially expressed His-tagged input cell lysates of Dsx^CR^ and various domain deletions of Dsx. Samples were tested with GST alone, GST-AbdB-N (C′) and various GST-tagged domain deletions of AbdB-N (D). Pulldown shows that AbdB-N and Dsx^CR^ interact through their HD (lanes 13-17) and DM domain (lanes 6-9), respectively. (C″-D″) SDS-PAGE stained with Coomassie Blue depicts almost equal loading of the GST-tagged protein samples.

Because Dsx^F^ promotes apoptosis, whereas Dsx^M^ blocks apoptosis of Dsx^+^tNBs (Birkholz et al., 2013a), we tested whether Dsx^F^ could induce *F3B3-lacZ* in the anterior CNS. We observed that ectopic expression of Dsx^F^ in mid-L2 (Fig. S2F for TS protocol) induced *F3B3-lacZ* expression in NBs of the anterior CNS 7 h later (Fig. 4A versus 4B, *n*=14 VNCs, *N*=3; Fig. 4C-C″, shown as inset to Fig. 4B-B″). Expectedly, many of the thoracic NBs expressing Dsx^F^ eventually underwent apoptosis in late L3 (Fig. S2B for TS protocol; Fig. 4D versus 4E), as judged by comparison of the number of GFP-marked surviving thoracic lineages in L3 (50.14±0.6 NBs, *n*=14 VNCs, *N*=2; Fig. 3C) compared with controls (144.6±2.5 NBs *n*=17, *N*=2; Fig. 3C). In contrast, ectopic expression of Dsx^M^ did not result in any NB apoptosis in the thoracic region (152.0±4.1 NBs, *n*=9 VNCs, *N*=2; Fig. 3C, Fig. 4D versus 4F). In fact, Dsx^M^ repressed the background expression of *F3B3-lacZ* in the anterior CNS (Fig. 4F″). Dsx^M^ also repressed developmental apoptosis as well as *F3B3-lacZ* expression in larval abdominal NBs (yellow arrowhead in Fig. 4G-G″″; *n*=12 VNCs, *N*=3). To test the role of the female-specific C-terminal region in apoptosis, we ectopically expressed a Dsx form that lacked the entire female-specific region and contained only the Dsx common region (hereafter referred to as Dsx^CR^; Fig. 5A). Interestingly, we observed that Dsx^CR^ expression was capable of causing thoracic NB apoptosis in late L3 (57.50±1.9 NBs, *n*=12 VNCs; Fig. 3C, Fig. 4D versus 4H) with an efficiency almost comparable to that of Dsx^F^ (50.14±0.6 NBs, *n*=14; Fig. 3C). We also observed that Dsx^CR^ was capable of causing the death of all four Dsx^+^tNBs in the male VNC (*n*=18 VNC; Fig. S2A for TS protocol) and ectopically inducing *F3B3-lacZ* in the anterior CNS (Fig. 4D″ versus 4H″).

These results indicated that the common region of Dsx protein is sufficient to bring about NB apoptosis, and that the female-specific region complements this capacity whereas the male-specific region seems to inhibit it.

### AbdB and Dsx^F^ are necessary for apoptosis

Our results suggest that both AbdB and Dsx^F^ are capable of inducing tNB apoptosis. Because AbdB seems to positively regulate Dsx in NBs, we wanted to know whether both AbdB and Dsx^F^ are necessarily required for Dsx^+^tNB apoptosis or whether AbdB executes apoptosis solely by regulating Dsx^F^ expression in tNBs. To test this, we induced a simultaneous RNAi-mediated knockdown of AbdB and overexpression of Dsx^F^ from early L1 and examined its effect in late L3 (Fig. S2A for TS protocol). We could successfully recover as many as 12 Dsx-expressing NBs (Fig. 4I-I″″) in the terminal region of female VNCs in late L3 (*n*=8 VNCs, *N*=2) compared with controls (expressing just Dsx^F^), which had none. This implied that even though Dsx^F^ was expressed in NBs, knockdown of AbdB was sufficient to block their cell death and both AbdB and Dsx^F^ are needed for NB apoptosis in the terminal region of female VNCs.

### AbdB and Dsx^CR^ interact *in vitro*

Next, we decided to test the physical interaction between AbdB and Dsx. We started out by using the Dsx common region (Dsx^CR^) and tested its interaction with AbdB (Fig. 5A). Both morphogenetic (AbdB-m) and regulatory (AbdB-r) isoforms of AbdB are expressed in the CNS (Birkholz et al., 2013b). Because Dsx^+^tNBs reside in PS13, which mainly expresses the AbdB-r isoform, as well as for ease of handling, we chose a truncated version of AbdB-r for our biochemical experiments that lacks the first 89 amino acids (hereafter referred to as AbdB-N; Fig. 5B).

In a GST-pulldown assay, we observed that although GST alone did not pull down His-Dsx^CR^ (lane 2, Fig. 5C′ and lane 12, Fig. 5D), GST-AbdB-N could successfully pull down His-Dsx^CR^ (approximately 55 kDa; lane-6, Fig. 5C′ and lane 13, Fig. 5D). This suggested that Dsx and AbdB interact with each other *in vitro* and that the interaction is mediated through a domain lying outside the sex-specific region of the protein. Next, we made a series of truncations for both Dsx and AbdB to map the respective interaction domains (detailed in Fig. 5A,B and Fig. S6B,C). We found that although individual deletions of the N-terminal domain (ΔN-DsxCR) or the dimerization domain (ΔDD-Dsx^CR^) of Dsx did not alter its binding with AbdB (lanes 7 and 9, Fig. 5C′), deletion of the DM domain or the DNA-binding domain (ΔDBD-Dsx^CR^, 41-101 amino acids) completely abolished the interaction between the two proteins (lane 8, Fig. 5C′). Reciprocal mapping of the domain within AbdB responsible for its interaction with Dsx^CR^ was carried out using four constructs centred around the AbdB HD region (60 amino acids of the HD and, additionally, two amino acids N-terminal to the HD and four C-terminal to the HD; Fig. 5B and Fig. S6C). For the ease of representation, we chose to call the entire stretch of 66 amino acids AbdB-HD (Fig. 5B). We found that AbdB-HD retained its ability to interact with Dsx^CR^ when the regions C-terminal and N-terminal to HD were deleted (lanes 15 and 16, Fig. 5D). However, deletion of AbdB-HD (AbdB-N-ΔHD) abolished its interaction with Dsx^CR^ completely (lane 17, Fig. 5D). Expectedly, AbdB-HD alone was sufficient to pull down His-Dsx^CR^ (lane 14, Fig. 5D). These results show that AbdB and Dsx interact with each other *in vitro* through the DM domain (DBD) of Dsx and the HD of AbdB.

### Dsx^CR^ and AbdB show cooperative binding on the apoptotic enhancer

**Fig. 6.**
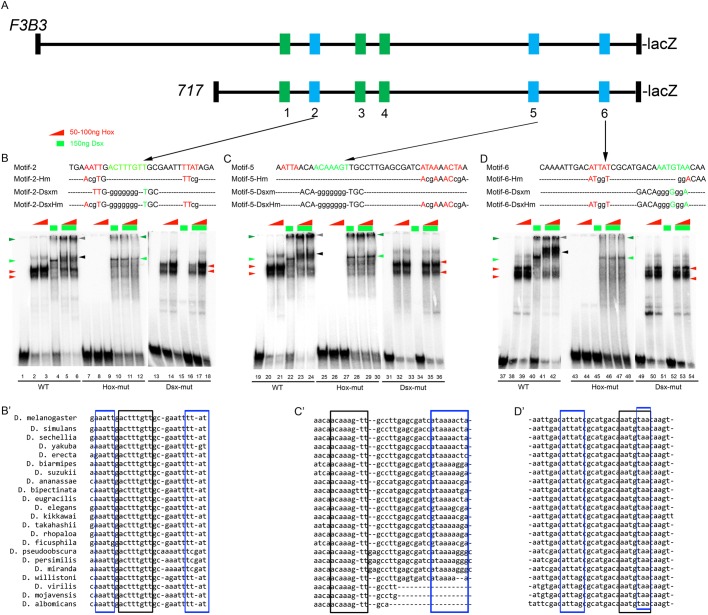
**Dsx^CR^ and AbdB cooperatively bind on the sex-specific apoptotic enhancer.** (A) Schematic of *F3B3-lacZ* and *717-lacZ* depicting six motifs containing Dsx and AbdB binding sites. Blue motifs exhibit cooperative binding of Dsx^CR^ and AbdB whereas green motifs do not (refer to Fig. S3). (B-D) EMSA for AbdB, Dsx^CR^ and AbdB-Dsx^CR^ binding on wild-type (WT), AbdB mutant and Dsx mutant oligos for motifs 2, 5 and 6. WT motif sequences are colour-coded for Hox (red) and Dsx (green) binding sites. Mutant variants are shown in lower case. Red arrowheads indicate Hox–DNA complex; green and dark green arrowheads indicate Dsx monomer and dimer complexes with DNA, respectively. Black arrowhead indicates Hox–Dsx–DNA cooperative complex. Grey arrowhead indicates cooperative complex with two molecules of Dsx. (B′-D′) Sequence comparison across multiple species for three motifs analysed. Dsx and Hox binding sites are highlighted by black and blue boxes, respectively.

To test whether the interaction between Dsx^CR^ and AbdB also took place on the DNA, we analysed the 1 kb enhancer for potential Dsx and AbdB binding sites. We identified 11 potential Dsx sites conforming to the variation of the consensus sequence (RNNACWAWGTNNY) (Luo et al., 2011; Clough et al., 2014). All these sites had AT-rich sequences (potential Hox binding sites) within 20 bp proximity (Sorge et al., 2012). Six out of eleven motifs showed Dsx^CR^ binding by EMSA (Fig. 6 and Fig. S3). These six Dsx-binding motifs were concentrated in a highly conserved 717 bp subfragment of the 1 kb apoptotic enhancer (motifs 1-6, Fig. 6A). Three of the six binding motifs (motifs 2, 5 and 6) were found to be highly conserved (Fig. 6B′-D′) and were analysed in detail.

We used two different concentrations of Hox protein AbdB (50 and 100 ng) and a fixed concentration of Dsx^CR^ (150 ng) and found that these proteins bound individually to motif 2 (lanes 2-4, Fig. 6B), motif 5 (lanes 20-22, Fig. 6C) and motif 6 (lanes 38-40, Fig. 6D). Dsx protein is known to have a dimerization domain and therefore Dsx binds on DNA both as a monomer and dimer. The specificity of the binding for AbdB and Dsx was established using oligos mutant for potential AbdB and Dsx binding sites. We found that an oligo mutant for the Hox binding site resulted in loss of AbdB binding for all three binding motifs (lanes 8-9, 26-27 and 44-45; Fig. 6B-D), leaving the Dsx binding either intact in the case of motif 5 or partly affected in the case of motifs 2 and 6 (lanes10, 28 and 46, Fig. 6B-D). Reciprocally we found that an oligo mutant for potential Dsx binding sites showed a complete loss of Dsx^CR^ binding (lanes 16, 34 and 52, Fig. 6B-D) leaving AbdB binding mostly unaltered (lanes 14-15, 32-33 and 50-51, Fig. 6B-D) in all three motifs. Interestingly, we found that AbdB protein seems to form a cooperative complex of slower mobility in the presence of Dsx^CR^, as indicated by the black arrowhead in Fig. 6B-D and by an increase in binding intensity of the Dsx^CR^ dimer band (grey arrowhead), which was observed for all three motifs (lanes 5-6, 23-24, 41-42, Fig. 6B-D). This cooperative synergism of the two proteins is lost in the case of oligos with mutation for either AbdB (lanes 11-12, 29-30 and 47-48, Fig. 6B-D) or Dsx binding sites (lanes 17-18, 35-36 and 53-54, Fig. 6B-D).

These results show that AbdB and Dsx^CR^ cooperatively interact with each other on three binding motifs (motifs 2, 5 and 6) found on the sex-specific apoptotic enhancer and their interaction on DNA is probably mediated through the HD of AbdB and the DM domain of Dsx.

### Dsx^F^ helps AbdB to select and activate the apoptotic enhancer

**Fig. 7.**
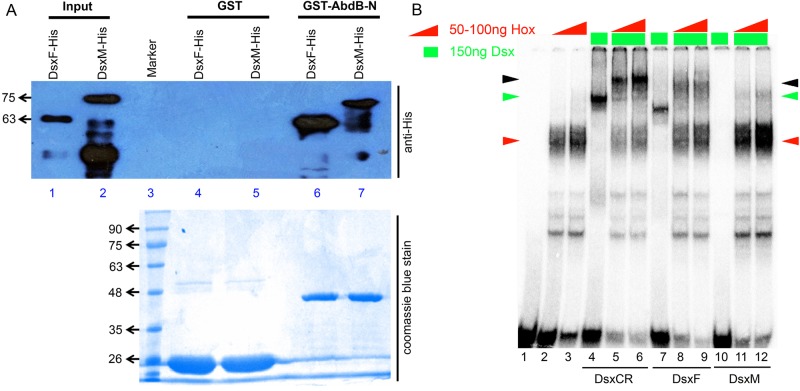
**Sex-specific interaction of Dsx with AbdB.** (A) Western blot of the GST pulldown shows that Dsx^F^ interaction with AbdB is stronger than that of Dsx^M^. Bacterially expressed His-tagged input cell lysates of Dsx^F^ and Dsx^M^ (lanes 1 and 2) are pulled down by GST-AbdB (lanes 6 and 7) but not by GST alone (lanes 4 and 5). SDS-PAGE stained with Coomassie Blue depicts almost equal loading of the GST-tagged protein samples. (B) EMSA for Dsx^CR^, Dsx^F^ and Dsx^M^ with AbdB on motif 5 shows that Dsx^CR^ and Dsx^F^ bind cooperatively with AbdB and Dsx^M^ does not. Red arrowheads indicate Hox–DNA complex, green arrowheads indicate Dsx–DNA complex, black arrowhead indicates Hox–Dsx–DNA cooperative complex.

Because Dsx^M^ represses apoptosis of abdominal NBs (Fig. 4G′), we decided to test the isoform-specific interaction of Dsx with AbdB. For this, AbdB was tested for its interaction with full-length male- and female-specific isoforms of Dsx in a GST pulldown assay. Here, we observed that although GST alone did not pull down either of the isoforms (lanes 4 and 5, Fig. 7A), GST-tagged AbdB-N could successfully pull down both Dsx^F^ and Dsx^M^ (approximately running at 63 and 75 kDa, respectively; lanes 6 and 7, Fig. 7A). We observed that AbdB interacted with Dsx^F^ much more strongly than with Dsx^M^. Because Dsx^+^tNB apoptosis and enhancer expression is female-specific, we tested AbdB and Dsx^F^ for cooperative interaction on motif 5. We observed that although AbdB (lanes 2 and 3, Fig. 7B), Dsx^CR^ (lane 4, Fig. 7B) and Dsx^F^ (lane 7, Fig. 7B) bound individually to DNA, there was no significant binding of Dsx^M^ (lane 10, Fig. 7B) on the DNA. In the presence of AbdB, we observed a lower mobility cooperative complex for both Dsx^F^ and Dsx^CR^ (lanes 5-6 and 8-9, Fig. 7B), but we did not observe a significant band of similar or lower mobility for Dsx^M^, considering its larger size (lanes 11 and 12, Fig. 7B), although we did observe an increase in AbdB band intensity. These results suggest that although Dsx^CR^ and Dsx^F^ cooperatively interact with AbdB to bind on DNA, Dsx^M^ does not (Fig. 7B). The results also suggest that the Dsx^CR^ region is capable of forming a complex with AbdB on the apoptotic motifs of the enhancer; furthermore, the female-specific region facilitates complex formation but the male-specific region seems to interfere with the same.

These results support the apoptosis-inducing potential of the Dsx isoforms tested earlier. Dsx^F^ (and Dsx^CR^), which formed a cooperative complex with AbdB on the apoptotic enhancer, were capable of inducing apoptosis of Dsx^+^tNBs in females whereas Dsx^M^, which does not show cooperation with AbdB on the apoptotic enhancer, repressed the death of Dsx^+^tNBs in females.

This also suggests that Dsx isoforms can act as a sex-specific cofactor for Hox gene *Abd-B* and can help it to select its target genes.

### Dsx^F^ is required for activity of the apoptotic enhancer

**Fig. 8.**
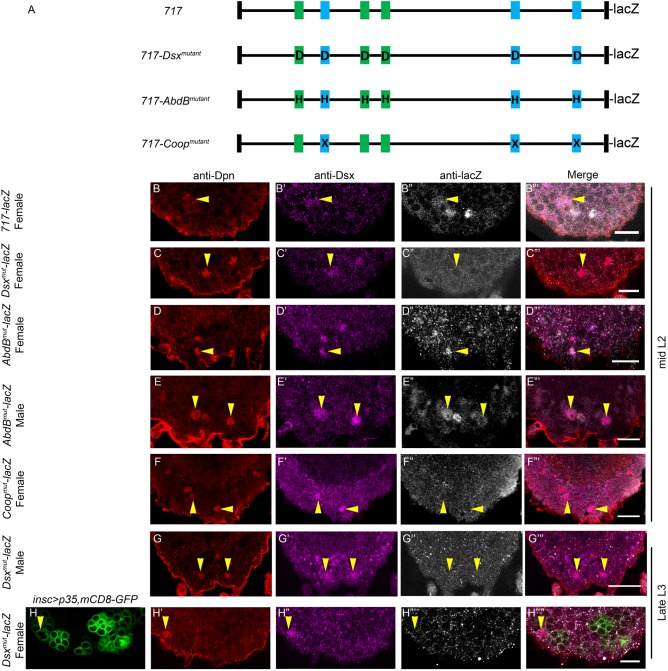
**Enhancer mutagenesis analysis *in vivo*.** (A) Schematic of *717-lacZ*, *717-Dsx^mutant^-lacZ*, *717-AbdB^mutant^-lacZ* and *717-Coop^mutant^-lacZ.* (B-F) Shows the *enhancer-lacZ* expression for wild-type and various mutant versions of the enhancer in Dsx^+^tNBs at mid-L2. *717-lacZ* expresses normally (*n*=21) (B″) but *Dsx^mutant^-lacZ* (*n*=14) (C″) and *Coop^mutant^-lacZ* (*n*=12) (F″) fail to express in Dsx^+^tNBs of female VNCs in mid-L2. *AbdB^mutant^-lacZ* expression is unaffected in Dsx^+^tNBs of females (*n*=9) (D″), but male VNCs show ectopic expression in Dsx^+^tNBs where it is normally not expressed (*n*=11) (E″). In contrast, *Dsx^mutant^-lacZ* expression is not observed in either male (*n*=15) (G-G‴) or cell death-blocked female VNCs (*n*=8) (H-H″″) in late L3 (Fig. S2A for TS protocol). *n* indicates the number of VNCs analysed. Yellow arrowheads indicate Dsx^+^tNBs. Scale bars: 10 µm. All the images are single confocal sections.

To test the *in vivo* relevance of the binding sites, we mutagenized six Dsx binding sites that bound Dsx^CR^ (*717-Dsx^mutant^-lacZ;* mutations as shown in Fig. 6B-D, and supplementary Materials and Methods). We tested the activity of the mutagenized enhancer in mid-L2 in Dsx^+^tNBs of the female VNC. Although we observed the expression of *717-lacZ* in Dsx^+^tNBs of the female VNC (Fig. 8B-B‴; *n*=21 VNCs, *N*=4), we could not observe any *lacZ* expression in the case of *717-Dsx^mutant^-lacZ* at the same time in these cells (Fig. 8C-C‴). To rule out any delay in enhancer initiation, we tested *717-Dsx^mutant^-lacZ* for its expression in late L3 by temporally blocking Dsx^+^tNB apoptosis using p35 (Fig. S2A for TS protocol). We found that *717-Dsx^mutant^-lacZ* did not show any expression in the Dsx^+^tNBs of female VNCs in late L3 (Fig. 8H-H″″). In contrast to what is known for other Dsx target genes, we observed that *717-Dsx^mutant^-lacZ* did not show any ectopic expression of *lacZ* in Dsx^+^tNBs of the male VNC (Fig. 8G-G‴). This suggests that Dsx^M^-mediated repression of apoptosis and *enhancer-lacZ* (Fig. 4G) is through a mechanism other than simple Dsx^M^ binding on the enhancer and silencing. This also suggests that the Dsx binding motif in the enhancer is crucial for its activation in the female VNC, implying a role for Dsx^F^ in sex-specific enhancer initiation.

### AbdB acts as a repressor of the apoptotic enhancer

Next, AbdB binding sites were mutagenized across the six motifs, leaving neighbouring Dsx binding sites intact (*717-AbdB^mutant^-lacZ;* mutations as shown in Fig. 6B-D, see supplementary Materials and Methods for details). We found that reporter *lacZ* expression in the case of *717-AbdB^mutant^-lacZ* in the female VNC at mid-L2 was unaffected in Dsx^+^tNBs (Fig. 8D-D‴). Interestingly, in the case of male VNCs, we found that reporter *lacZ* was activated in Dsx^+^tNBs (Fig. 8E-E‴). This suggested that AbdB binding on the enhancer normally keeps it in a repressed state.

Because Dsx^CR^ and Dsx^F^ cooperate with AbdB on three motifs (motifs 2, 5 and 6) in the enhancer and Dsx^M^ does not, we hypothesized that these motifs are central to sex-specific activation of the apoptotic enhancer and, therefore, cell death in female VNCs. To test this, we mutagenized both Dsx and AbdB binding sites in these three cooperative motifs (*717-Coop^mutant^-lacZ;* mutations as shown in Fig. 6B-D)*.* Mutagenesis of AbdB-Dsx binding sites found in these motifs was sufficient to abrogate enhancer activity in Dsx^+^tNB in the female VNC (Fig. 8F-F‴). This underlined the importance of the three motifs and the cooperative interaction of AbdB-Dsx on these motifs for sex-specific activation of the enhancer.

These results show that AbdB normally functions as a repressor of the apoptotic enhancer. Perhaps its capacity to form a cooperative complex with Dsx^F^ converts it into an activator in the complex, and it therefore plays a central role in sex-specific activation of the apoptotic enhancer.

### Dsx is the trigger for apoptosis

**Fig. 9.**
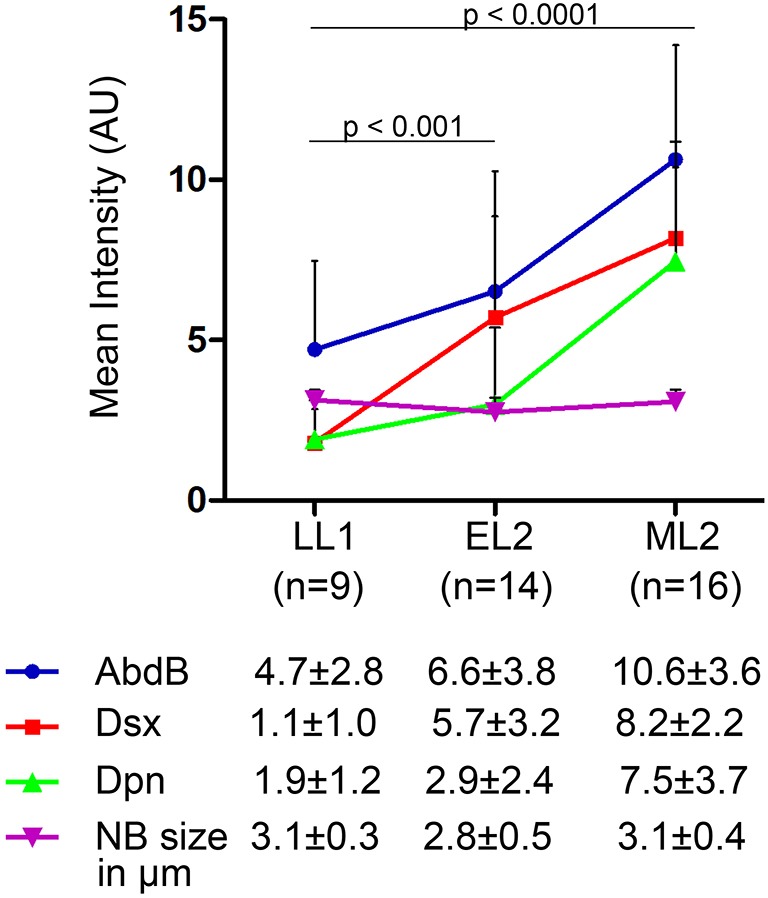
**Dsx^F^ act as the trigger for Dsx^+^tNBs apoptosis in female VNCs.** Graph showing increasing levels of Dsx, AbdB and Dpn staining along with NB size, across different stages. *n* number of VNCs analysed. Significance (*P*-value) was calculated using the two-tailed Student's paired *t*-test for Dsx levels calculated across three stages.

An enhancer mutant for Dsx binding sites failed to initiate the expression of the reporter gene, and AbdB alone functions as a repressor of the wild-type enhancer. Therefore, we explored whether Dsx^F^ can act as a trigger for activation of apoptosis in females by quantifying Dsx levels using antibody staining from late L1 to mid-L2 (temporal window prior to apoptosis). Because it is difficult to sex the larvae at L1, quantification was carried out for a mixed population of male and female larvae. We observed a steady increase in levels of Dsx across these stages (with almost no expression in late L1), although the size of the cells remained almost constant (Fig. 9). A simultaneous increase in AbdB expression levels was also observed in the same time window. This led us to propose that levels of Dsx increase in both sexes, but this acts as a trigger for apoptosis of Dsx^+^tNBs only in females.

## DISCUSSION

Multicellular organisms are known to be a mosaic of cells and tissues, which are on a “need to know basis” as far as sex is concerned (Hempel and Oliver, 2007; Lee et al., 2002; Rideout et al., 2010; Robinett et al., 2010). These cells and tissues have to tightly regulate the expression of sex-specific genes and their dimorphic targets such as *Lgr3* (Meissner et al., 2016), *dsx* (Zhou et al., 2014), *bab1* (Williams et al., 2008), Yp genes (An and Wensink, 1995; Burtis et al., 1991; Coschigano and Wensink, 1993) and *fmo-2* (Luo and Baker, 2015); more so because their expression might be required in one tissue (or sex) and detrimental in another (Clough et al., 2014). Sexually dimorphic enhancers are thought to function as genetic switches (Tanaka et al., 2011; Williams and Carroll, 2009) and are central to such tight regulation. They are expected to have binding sites for both sex-specific and tissue- or region-specific TFs. Enhancers for the yolk protein (Yp) genes (An and Wensink, 1995; Burtis et al., 1991; Coschigano and Wensink, 1993), *bab1* (Kopp et al., 2000; Williams et al., 2008) and the RHG genes (required for female-specific Dsx^+^tNB apoptosis) are the only known examples of this kind. In Dsx^+^tNBs, we observed that AbdB and Dsx work in hierarchical as well as collaborative fashion, as reported earlier for non-neural tissues (Wang et al., 2011; Wang and Yoder, 2012; Kopp et al., 2000; Tanaka et al., 2011; Williams et al., 2008). We found that these two proteins physically interact through the highly conserved HD (of AbdB) and DM domain (of Dsx) (Fig. 5). AbdB and Dsx also show cooperative interaction on the sexually dimorphic apoptotic enhancer (Fig. 6), which has not been reported earlier (Williams et al., 2008). This identifies an unexpected novel functional link between the two kinds of TFs and underlines their capacity to use each other as partner or cofactor to influence specific outcomes in male and female bodies. Because Dsx^+^tNBs apoptosis is specific to terminal segments of the female VNC, it is suggested that context and interaction of AbdB and Dsx^F^ on the enhancer ensures that RHG genes are activated only in females (and not in males, or in NBs of other regions of the developing CNS).

Our results also suggest that although the Dsx common region is crucial for DNA binding and its interaction with AbdB (for target gene selection), the sex-specific regions have minimal impact on the same. In fact, the latter seem to play an important role in determining the regulatory output of the AbdB–Dsx complex, depending on the context (target gene, cell type and sex). Therefore, it is equally plausible to expect that Dsx^M^ and AbdB also cooperate in selection and regulation, albeit on a different set of target genes. This is in agreement with the finding that both Dsx^F^ and Dsx^M^ have a similar genomic occupancy pattern and therefore have many common target genes, yet many of them have tissue- and sex-specific expression (Clough et al., 2014). We propose that this could be a consequence of their interaction with spatial TFs such as AbdB or other HD-containing factors (including other Hox genes). Insights from mutagenesis studies on the Yp gene fat body enhancer element (*o-r*) support this idea (Coschigano and Wensink, 1993), wherein Dsx has been suggested to work with spatial TFs (such as AEF-1 and C/EBP) to localize Yp gene expression to the female fat body. In the fat body, Dsx^F^ was suggested to synergize with a C/EBP class of TFs to activate Yp genes, whereas the C-terminal region of Dsx^M^ was thought to interfere with this complex formation and hence its activation (Coschigano and Wensink, 1993).

Enhancer mutagenesis and the Dsx expression profile prior to apoptosis imply a role of Dsx^F^ in female-specific activation of RHG genes in Dsx^+^tNBs. However, a relative delay in the expression of Dsx compared with AbdB suggests a role for additional regulation. The temporal series TF Castor (Isshiki et al., 2001) has been shown to play a role in abdominal NBs apoptosis through regulation of the *grh* gene (Maurange et al., 2008). However, we did not find Grh protein to be crucial for Dsx^+^tNB apoptosis, raising the possibility of Dsx being a direct target of Castor.

Considering the literature, we expected that Dsx^M^ binding on the enhancer would actively silence apoptosis in males (Luo and Baker, 2015; Coschigano and Wensink, 1993). Interestingly, it was the mutagenesis of AbdB binding sites that activated the enhancer in males. This implies that AbdB (and not Dsx^M^) acts as a repressor for the wild-type enhancer. It also suggests that the capacity of AbdB to form a cooperative complex with Dsx^F^ perhaps converts the two into an activator complex on the death enhancer (exclusively in females). In thoracic NBs that lack Dsx expression, misexpression of Hox genes causes NB apoptosis (Bello et al., 2003); we believe that Grh plays an important role in this apoptosis (Khandelwal et al., 2017).

The cellular role of Hox genes (like any other TF) is expected to be highly context dependent. For instance, Hox genes have been shown to directly regulate RHG genes to function both as promoters and repressors of apoptosis in different contexts (Bello et al., 2003; Miguel-Aliaga and Thor, 2004; Lohmann et al., 2002). More specifically, the role of AbdB in the survival of dMP2 and dMP1 neurons in embryonic VNC (Miguel-Aliaga and Thor, 2004) is most likely executed by direct binding and repression of a common neuronal enhancer for *grim* and *reaper* genes. Similarly, female-specific motor neurons expressing FS-Ilp7 could also be using AbdB for their selective survival, or for their male-specific death. It is of interest to check the former cell types for Dsx expression and their possible cooperative role with AbdB to repress RHG genes in both sexes; however, the sex-specific survival of motor neurons expressing FS-Ilp7 is more intriguing, wherein the detailed mechanism of male-specific apoptosis that occurs in the absence of Dsx expression is yet to be elucidated (Garner et al., 2018).

Even though abdominal NBs and Dsx^+^tNBs employ the same enhancer for apoptosis, they use different molecular strategies. In abdominal NBs, a pulse of AbdA in mid-L3 triggers apoptosis (Bello et al., 2003) and requires Grh, Exd and Notch signalling (Khandelwal et al., 2017). Dsx^+^tNBs, however, utilize increasing levels of Dsx^F^ as a trigger to recruit otherwise repressive AbdB into a cooperative activator complex specifically in females but not in males (Fig. S7). This is a clever modification of the abdominal apoptotic strategy by replacing Grh, Notch and Exd with Dsx to engineer the cell death of a desired cell type in females at an earlier time point. Because there is a considerable overlap between Dsx and DMRT1 target genes (Murphy et al., 2010), utilization of HD-containing TFs (as cofactors) can be a general strategy used by both Dsx and DMRT1 to carry out their tissue-, cell type- and sex-specific gene regulations.

## MATERIALS AND METHODS

### Fly stocks and fly husbandry

Fly stocks used were *F3B3-lacZ*, *717-lacZ*, *M22/TM6Tb*, *FRT-exd^1^* (Khandelwal et al., 2017); *dsx-GAL4* (S. F. Goodwin, University of Oxford, UK) (Rideout et al., 2010); *MM3/TM6Tb* and *grim^A6C^/TM6Tb* (Kristin White, Harvard Medical School, USA) (Tan et al., 2011); *FRT-AbdB^M1^* (E. Sanchéz-Herrero, CSIC-UAM, Spain); *grh^B37^/CyO* and *grh^370^*/*CyO* (Sarah Bray, University of Cambridge, UK) (Almeida and Bray, 2005); *hsflp*, *FRT19A*, *tub-GAL80*; *tub-GAL4*, *UAS-mCD8-GFP*/*CyO*-GFP (H. Reichert, University of Basel, Switzerland); *UAS-dcr2; inscGAL4 UAS-mCD8-GFP; tub-GAL80^ts^* (J. A. Knoblich, IMBA, Austria) (Neumüller et al., 2011); *UAS-Dsx^M^*, *UAS-Dsx^F^* [G. Lee, University of Tennessee, USA, and Bloomington *Drosophila* Stock Center (BDSC), 44224 and 44223] (Lee et al., 2002); *Canton-S* (BDSC, 64349); *UAS-Dsx^CR^*-DPiM (K. VijayRaghavan, NCBS, India) (Guruharsha et al., 2011); *ix^1^* (BDSC, 205); *ix^2^* (BDSC, 372); *UAS-p35* [*Drosophila* Genomics Resource Center (DGRC), 108019]; *UAS-AbdB* (DGRC, 106120); *elav[C155]-GAL4*, *UAS-mCD8-GFP*, *hsflp1*, *w* (BDSC, 5146); *yw*; FRT82B *tub-GAL80*-LL3 (BDSC, 5135); *Notch*-*RNAi* (BDSC 28981); *AbdB*-*RNAi* [Vienna *Drosophila* Resource Center (VDRC), 11024]; and *hth-RNAi* (NIG-17117-R4 and R2).

The following transgenic lines were used: *717-Dsx^mutant^-lacZ*, *717-AbdB^mutant^-lacZ* and *717-Coop-^mutant^-lacZ*, generated by site-specific insertion at attP40-25C6. The *rpr^17^* deletion line was generated by mobilization of the P-element, inserted 891 bp from the 5′-region of *rpr* transcript (BDSC-14326) [the deletion line was confirmed through genomic PCR mapping (Fig. S1)]. A recombinant strain of *grim^A6C^-rpr^17^/TM6B,Tb* was obtained after screening 20 independent recombinant lines for block of abdominal and terminal pNB apoptosis.

All the fly stocks and crosses were maintained at 25°C unless otherwise stated. For fly crosses, 4 h egg collections were carried out and the larvae reared at 25°C. All larvae were dissected at the desired larval stage.

### Immunohistochemistry and image acquisition

Larvae of the desired sex, genotype and age were dissected as described earlier (Khandelwal et al., 2017) with the following variations: fixation was done for 30 min at room temperature and immunostaining with primary antibodies overnight at 4°C. The primary antibodies used were rabbit anti-Dpn (1:5000) (Khandelwal et al., 2017), mouse anti-Grh (1:2000) (Khandelwal et al., 2017), mouse Abd-B (1:20; 1A2E9, DSHB), rat anti-Dsx (1:3000; Fig. S6F), mouse anti-Exd (1:20; B11M, DSHB), mouse anti-NICD (1:50; C17.9C6, DSHB), guinea pig anti-Hth (1:100; GP52, Richard Mann), chicken anti-β-gal (1:2000; ab9361, Abcam). For the anti-Dsx antibody, N-terminal His-tagged Dsx^CR^ fusion protein [amino acid (aa) residues 1-397] (Fig. 5A) was used to raise the antibody at the in-house animal facility.

All brain samples were mounted in 70% glycerol and fluorescent images acquired using a Zeiss LSM 700 inverted confocal microscope and analysed using ZEN 2012 software. The pNBs were counted by scanning the entire scans in the region of interest while taking care that no cell was counted twice. The images were processed using ImageJ and Adobe Photoshop CS2 software. For all quantifications, the Student's unpaired *t*-test was used to assess significance, except for Fig. 9A where the Student's paired *t*-test was used (GraphPad Prism software).

### Temperature shift experiments

Fly crosses were set up at 18°C between virgin females of the genotype *UAS-dcr2; inscGAL4-UASmCD8-GFP; tub-GAL80^ts^* with males of genotypes *UAS-p35*; *UAS-AbdB-RNAi*; *UAS-Notch-RNAi*; *UAS-Hth-RNAi*; *UAS*-*Dsx^F^*; *UAS-Dsx^M^*; *F3B3-lacZ, UAS-Dsx^F^*; *F3B3-lacZ, UAS-Dsx^M^*; *UAS-Dsx^CR^-DPiM*; *F3B3-lacZ, UAS-Dsx^CR^-DpiM; UAS-AbdB*; *UAS-AbdB, F3B3-lacZ*; and *UAS-AbdB-RNAi, UAS-Dsx^F^* with suitable controls, as detailed in the genotype section. Egg collections were carried out at 4 h and the larvae reared at 18°C until the desired stage (time) and were then shifted to 30°C. The larvae were sexed, separated at specific times and subsequently dissected according to the requirement of each individual experiment. Six different TS protocols were employed, as described in Fig. S2A-F. The specific protocol used in each experiment is indicated by the figure number. The *lac-Z* levels were quantified for Dsx^+^tNBs as described previously (Khandelwal et al., 2017).

### Mosaic Analysis of Cell repressible marker (MARCM)

MARCM clones were generated as described previously (Lee and Luo, 2001) by crossing flies of relevant genotypes (detailed below). All the eggs collected for a duration of 4 h at 25°C were subjected to periodic heat shock of 1 h at 37°C after every 12 h, starting from the time of egg laying. Larvae were dissected at the late third instar larval stage. Dsx intensity was calculated as described above.

#### AbdB^M1^ clones

Females of the genotype *elav[C155]-GAL4, UASmCD8-GFP, hsflp1, w; FRT82B tub-GAL80-LL3* were crossed with males of genotype *FRT-AbdB^M1^/TM6B,Tb*.

#### *exd^1^* clones

Males of the genotype *hsflp*, *FRT19A*, *tubGAL80*; *tub-GAL4, UAS-mCD8-GFP*/*CyO-GFP* were crossed with females of *FRT- exd^1^*/*FM7*.

### Electrophoretic mobility shift assay

Electrophoretic mobility shift assays (EMSAs) were performed as described previously (Khandelwal et al., 2017). The following protein constructs were used for EMSA studies: N-terminal GST-tagged AbdB-N (aa residues 89-270) (Figs 6 and 7), N-terminal His-tagged Dsx^CR^ (aa residues 1-397) (Fig. 6), C-terminal His-tagged Dsx^F^ (aa residues 1-427), Dsx^M^ (aa residues 1-549) (Fig. 7). Dsx protein was purified and dialyzed in standard buffers containing 10 µM Zn^2+^SO_4_. All the binding reactions were set up in a 20 μl volume and incubated at room temperature for 40 min. EMSA images for Fig. 6B-D are a combination of multiple gels, as described in the legend. Each set of six lanes for wild-type, Hox mutant and Dsx mutant oligos are from the same gel. EMSA images in Fig. 7B, which compare the relative binding efficiencies of Dsx^CR^, Dsx^F^ and Dsx^M^, are from a single gel.

### GST pulldown experiments

The following constructs were used for the affinity pulldown assay: N-terminal His-tagged fusion proteins of Dsx^CR^ (aa residues 1-397), DsxΔN (aa residues 41-397), DsxΔDBD (aa residues 101-397) and DsxΔDD (aa residues 1-356); C-terminal His-tagged fusion proteins of Dsx^F^ (aa residues 1-427) and Dsx^M^ (aa residues 1-549); N-terminal GST-tagged fusion proteins were used for AbdB-N (aa residues 89-270), AbdB-HD (aa residues 162-228), AbdB-NΔCTD (aa residues 89-228), AbdB-ΔN (aa residues 162-270) and AbdB-NΔHD (aa residues 89-161 were deleted). Bacterial cultures were induced at OD_600_ 0.5 with 0.5 mM IPTG at 18°C for 2 h. The affinity pulldown experiments were performed as described in a previous report (Khandelwal et al., 2017). Representative blots from three repetition of the experiments are shown in Fig. 5 and Fig. 7.

### Genotypes analysed

Fig. 1B-B‴: *w/Y; UAS-mCD8-GFP/CyO; dsx-GAL4/dsx-GAL4*

Fig. 1C-C‴: *w/w; UAS-mCD8-GFP/+; dsx-GAL4/UAS-p35*

Fig. 1D-D‴: *UAS-Dcr2/w; inscGAL4, UAS-mCD8-GFP/+; tub-GAL80^ts^/UAS-p35*

Fig. 1E-E‴: *w/w; F3B3-lacZ/CyO; MM3/MM3*

Fig. 1F-F‴: *w/Y; UAS-nls-GFP/F3B3-lacZ; dsx-GAL4/+*

Fig. 1G-G‴: *w/w; UAS-nls-GFP/F3B3-lacZ; dsx-GAL4/UAS-p35*

Fig. 1H-H‴: *w/w; 717-lacZ/717-lacZ*

Fig. 1I-I″″: *UAS-Dcr2/w; inscGAL4, UAS-mCD8-GFP/717-lacZ; tub-GAL80^ts^/UAS-p35*

Fig. 2A-A‴: *elavGAL4, UAS-mCD8-GFP, hsFLP w/w; FRT82B tub-GAL80/ FRT82B-AbdB^M1^*

Fig. 2B-B‴: *hsflp, FRT19A, tub-GAL80/FRT19A-exd^1^; tubGAL4,UAS-mCD8-GFP/+*

Fig. 2C-C″:

*w/w; grim^A6C^-rpr^17^/grim^A6C^-rpr^17^* and *Canton S*

w/w; +/+; grim^A6C^/grim^A6C^

w/w; +/+; rpr^17^/rpr^17^

Fig. 2D:

Canton S

w/w; +/+; rpr^17^/rpr^17^

w/w; +/+; grim^A6C^/grim^A6C^

w/w; +/+; grim^A6C^-rpr^17^/grim^A6C^-rpr^17^

w/w; grh^B37/370^

UAS-dcr2/w; inscGAL4, UAS-mCD8-GFP/+; tub-GAL80^ts^/UAS-Hth-RNAi

w/w; ix^1/2^

UAS-dcr2/w; inscGAL4, UAS-mCD8-GFP/+; tub-GAL80^ts^/UAS-Notch-RNAi

Fig. 3A-A‴:

*elavGAL4, UAS-mCD8-GFP, hsFLP w/Y; FRT82B tub-GAL80/ FRT82B-AbdB^M1^* was the genotype of larvae dissected. White dotted cell is *FRT82B-AbdB^M1^/FRT82B-AbdB^M1^* and yellow dotted cell is *FRT82-AbdB^M1^/FRT82B- tub-GAL80* for 3rd chromosome.

Fig. 3B:

elavGAL4, UAS-mCD8-GFP, hsFLP w/Y; FRT82B tub-GAL80/FRT82B-AbdB^M1^

elavGAL4, UAS-mCD8-GFP, hsFLP w/Y; FRT82B tub-GAL80/FRT82B-AbdB^M1^

elavGAL4, UASmCD8-GFP, hsFLP w/w; FRT82B tub-GAL80/ FRT82B-AbdB^M1^

Fig. 3C:

UAS-Dcr2/Y; inscGAL4, UAS-mCD8-GFP; tub-GAL80^ts^/tub-GAL80^ts^

UAS-Dcr2/Y; inscGAL4, UAS-mCD8-GFP/UAS-AbdB; tub-GAL80^ts^/+

UAS-Dcr2/Y; inscGAL4, UAS-mCD8-GFP/+; tub-GAL80^ts^/UAS-Dsx^CR^

UAS-Dcr2/Y; inscGAL4, UAS-mCD8-GFP/+; tub-GAL80^ts^/UAS-Dsx^F^

UAS-Dcr2/Y; inscGAL4, UAS-mCD8-GFP/UAS-Dsx^M^; tub-GAL80^ts^/+

Fig. 3D:

UAS-Dcr2/w; inscGAL4, UAS-mCD8-GFP/F3B3-lacZ; tub-GAL80^ts^/UAS-p35

UAS-Dcr2/w; inscGAL4, UAS-mCD8-GFP/F3B3-lacZ; tub-GAL80^ts^/UAS-AbdB-RNAi

Fig. 3E-E″″: *UAS-Dcr2/Y; inscGAL4, UAS-mCD8-GFP; tub-GAL80^ts^/tub-GAL80^ts^*

Fig. 3F-F″″: *UAS-Dcr2/Y; inscGAL4, UAS-mCD8-GFP/UAS-AbdB; tub-GAL80^ts^/+*

Fig. 3G-G″″: *UAS-Dcr2/Y; inscGAL4, UAS-mCD8-GFP/+; tub-GAL80^ts^/F3B3-lacZ*

Fig. 3H-H″″: *UAS-Dcr2/Y; inscGAL4, UAS-mCD8-GFP/UAS-AbdB; tub-GAL80^ts^/F3B3-lacZ*

Comparison of surviving thoracic NBs in AbdB-overexpressing males and females was carried out for the following two genotypes:

Males: *UAS-Dcr2/Y; inscGAL4, UAS-mCD8-GFP/UAS-AbdB; tub-GAL80_ts_/+;*

Females: *UAS-Dcr2/w; inscGAL4, UAS-mCD8-GFP/UAS-AbdB; tub-GAL80_ts_/+*.

Fig. 4A-A′: *UAS-Dcr2/Y; inscGAL4, UAS-mCD8-GFP/F3B3-lacZ; tub-GAL80^ts^/+*

Fig. 4B-B″ and C-C″: *UAS-Dcr2/Y; inscGAL4, UAS-mCD8-GFP/F3B3-lacZ; tub-GAL80^ts^/UAS-Dsx^F^*

Fig. 4D-D″″: *UAS-Dcr2/Y; inscGAL4, UAS-mCD8-GFP/F3B3-lacZ; tub-GAL80^ts^/+*

Fig. 4E-E″″: *UAS-Dcr2/Y; inscGAL4, UAS-mCD8-GFP/F3B3-lacZ; tub-GAL80 ts/UAS-Dsx^F^*

Fig. 4F-G″″: *UAS-Dcr2/Y; inscGAL4, UAS-mCD8-GFP/UAS-Dsx^M^; tub-GAL80^ts^/F3B3-lacZ*

Fig. 4H-H″″: *UAS-Dcr2/Y; inscGAL4, UAS-mCD8-GFP/F3B3-lacZ; tub-GAL80^ts^/UAS-Dsx^CR^*

Fig. 4I-I″″: *UAS-Dcr2/w; inscGAL4, UAS-mCD8-GFP/+; tub-GAL80^ts^/UAS-Dsx^F^, UAS-AbdB-RNAi*

Fig. 8B-B‴: *w/w; 717-lacZ/717-lacZ; +/+*

Fig. 8C-C‴: *w/w; 717-Dsx^mutant^-lacZ/717-Dsx^mutant^-lacZ; +/+*

Fig. 8D-D‴: *w/w; 717-AbdB^mutant^-lacZ/ 717-AbdB^mutant^-lacZ; +/+*

Fig. 8E-E‴: *w/Y; 717-AbdB^mutant^-lacZ/717-AbdB^mutant^-lacZ; +/+*

Fig. 8F-F‴: *w/w; 717-Coop^mutant^-lacZ/717-Coop^mutant^-lacZ;+/+*

Fig. 8G-G‴: *w/Y; 717-Dsx^mutant^-lacZ/717-Dsx^mutant^-lacZ; +/+*

Fig. 8H-H″″: *UAS-Dcr2/w; inscGAL4, UAS-mCD8-GFP/Dsx^mutant^-lacZ; tub-GAL80^ts^/UAS-p35*

Fig. 9A: *w/w; UAS-mCD8-GFP/CyO; dsx-GAL4/ dsx-GAL4* and *w/Y; UAS-mCD8-GFP/CyO; dsx-GAL4/dsx-GAL4*

## Supplementary Material

Supplementary information
