# Supplementary material for: The Hox gene Abdominal-B uses DoublesexF as a cofactor to promote neuroblast apoptosis in the Drosophila central nervous system
Source: Development. 2019 Aug 22;146(16):dev175158. doi: 10.1242/dev.175158 (PMC6737903; doi:10.1242/dev.175158)
Supplement: Supplementary information [file develop-146-175158-s1.pdf]

Supplementary Materials and methods

717-Dsx^{mutant}-lacZ

Six Dsx binding sites which bound Dsx^{CR} were mutagenized while leaving neighbouring Hox binding sites intact (*717-Dsx^{mutant}-lacZ;* mutations as show in Fig-6B-D) in all of the five out of six motifs. In sixth motif where one of the Hox binding sites overlapped with Dsx binding site, we could map and kill the Hox binding by minimum perturbation, but same could not be done for the Dsx, hence entire Dsx binding site mutation affected one of the two Hox binding sites in the motif.

717-AbdB^{mutant}-lacZ

AbdB binding sites were mutagenized across the six motifs leaving neighbouring Dsx binding sites intact (*717-AbdB^{mutant}-lacZ;* mutations as show in Fig-6B-D) in all of the five out of six motifs. In sixth motif wherein one of the two Hox binding sites was found to be partly overlapping with Dsx binding site, we used a Hox mutation to the site which caused minimum possible perturbation to Dsx binding (Fig-6D).

<u>Genotypes used in Supplementary Figures:</u>

Fig-S1A-A”: *yw/w; M22/MM3*

Fig-S1B-B”: *w/Y; 717-lacZ/717-lacZ*

Fig-S4A-A”’, S4B-B”’, S4C-C””: *CantonS*

Fig-S4D-D’’: *w/Y; 717-lacZ*,

Fig-S4E-E’’; *w/w; 717-lacZ*

Fig-S4F- *w/w; 717-lacZ, w/Y; 717-lacZ*

Development • Supplementary information

Fig-S5A-A’: *w/w; grh*$^{B37/370}$*; worGAL4/UAS-mCD8-GFP*

Fig-S5B-B’: *hsflp, FRT19A, tub-GAL80/ FRT19A-exd*^{1}*; tubGAL4,UAS-mCD8-GFP/+*

Fig-S5C-C’: *UAS-dcr2/w; inscGAL4, UAS-mCD8-GFP/+; tub-GAL80*^{ts}*/UAS-Notch-RNAi*

Fig-S6A-A””: *UAS-Dcr2/w; inscGAL4, UAS-mCD8-GFP/+; tub-GAL80*^{ts}*/UAS-AbdB-RNAi, UAS-Dsx*^{F}

Fig-S6D-D”’: *w/Y; 717-Dsx*^{mutant}*-lacZ/717-Dsx*^{mutant}*-lacZ; +/+*

Fig-S6E-E”’: *w/w; 717-Coop*^{mutant}*-lacZ/717-Coop*^{mutant}*-lacZ; +/+*

Fig-S6F-F”’: *w/Y; UASmCD8-GFP/CyO; dsxGAL4/dsxGAL4*

Supplementary Figures

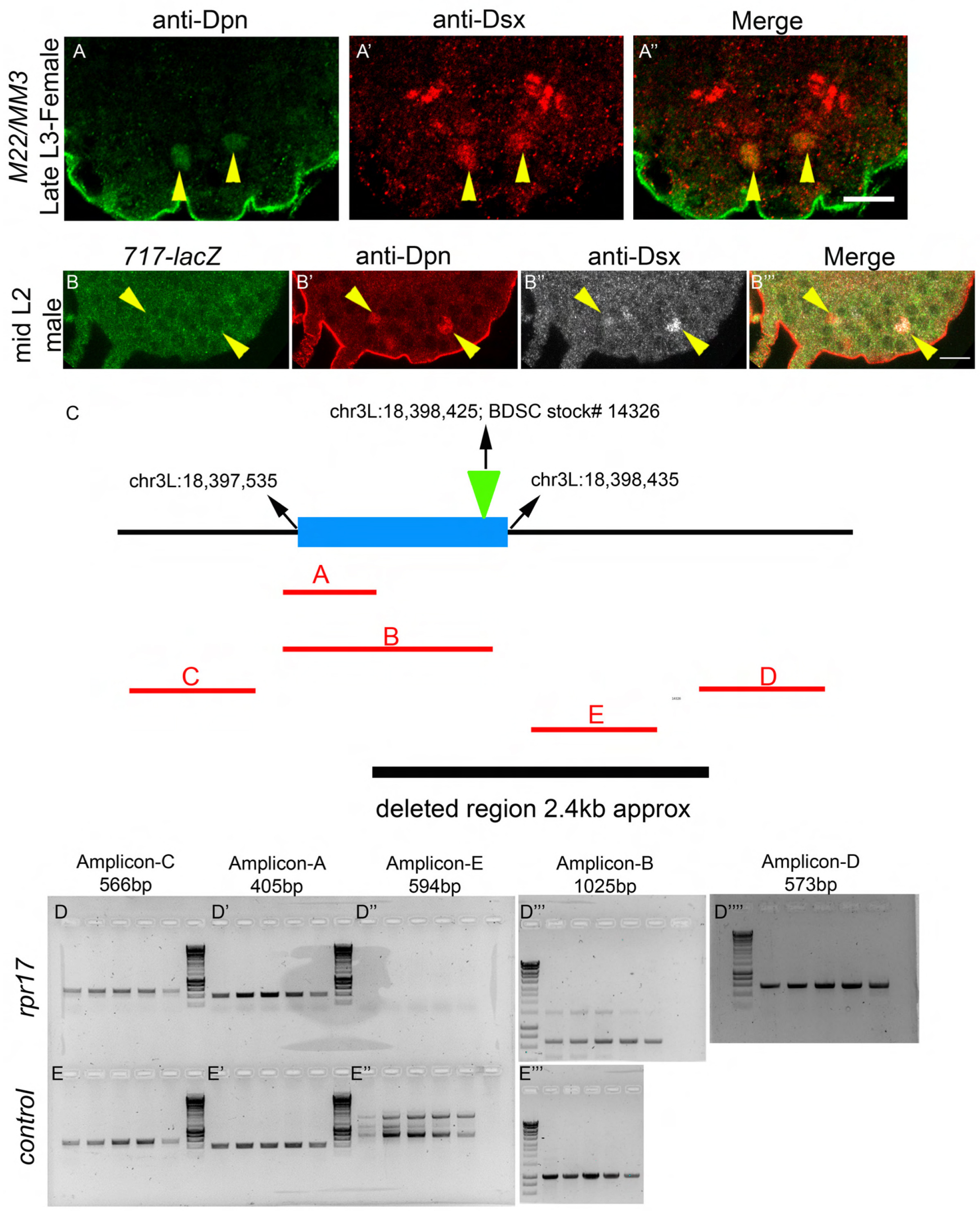

Development • Supplementary information

Figure-S1. Location of sex-specific apoptotic enhancer and mapping of *rpr*^{17} deletion. (A-A'') Dsx^{+}tNBs don't undergo apoptosis in *M22/MM3* heteroallelic deletion combination (n=14), suggesting that apoptotic enhancer for their death is located within 14.5 kb genomic region deleted in *M22*. (B) *717-lacZ* is not expressed in mid L2 stage in Dsx^{+}tNBs of male VNCs (n=21). Single confocal slice is shown. "n" indicates the number of VNCs. Scale bars is 10µm. Yellow arrowheads indicate Dsx^{+}tNBs. (C-E) Genomic mapping of the *rpr*^{17} deletion: A schematic representation genomic region surrounding the *rpr* transcript is shown. The green arrowhead marks the position of the P-element insertion (chr3L:18,398,425) with respect to 5' end of *rpr* transcript. The extent of genomic deletion was mapped by genomic PCR with *rpr*^{17} homozygous larvae. Approximately 2.4kb region between 3' end of amplicon-A and 5' end of amplicon-D is deleted (from Chr3L: 18,397,858 to 18,400,238; Release6). Approximate position and sizes of Amplicons are shown by red lines. Agarose gel pictures for genomic PCR for various amplicons in *Canton-S* and *rpr*^{17} homozygotes are shown Amplicons C (D-E), A (D'-E'), E (D''-E''), B (D'''-E'''), D (D''''). Amplicons-A, C, and D amplified in both *Canton-S* and *rpr*^{17}. Amplicon B and E did not amplify in *rpr*^{17}.

AmpliconA: Chr3L: 18,397,453...18,397,858 (405bp).

AmpliconB: Chr3L: 18,397,453...18,398,478 (1025bp).

AmpliconC: Chr3L: 18,396,640...18,397,206 (566bp).

AmpliconD: Chr3L: 18,400,238...18,400,811 (573bp)

AmpliconE: Chr3L: 18,399,028…18,399,622 (594bp)

Development • Supplementary information

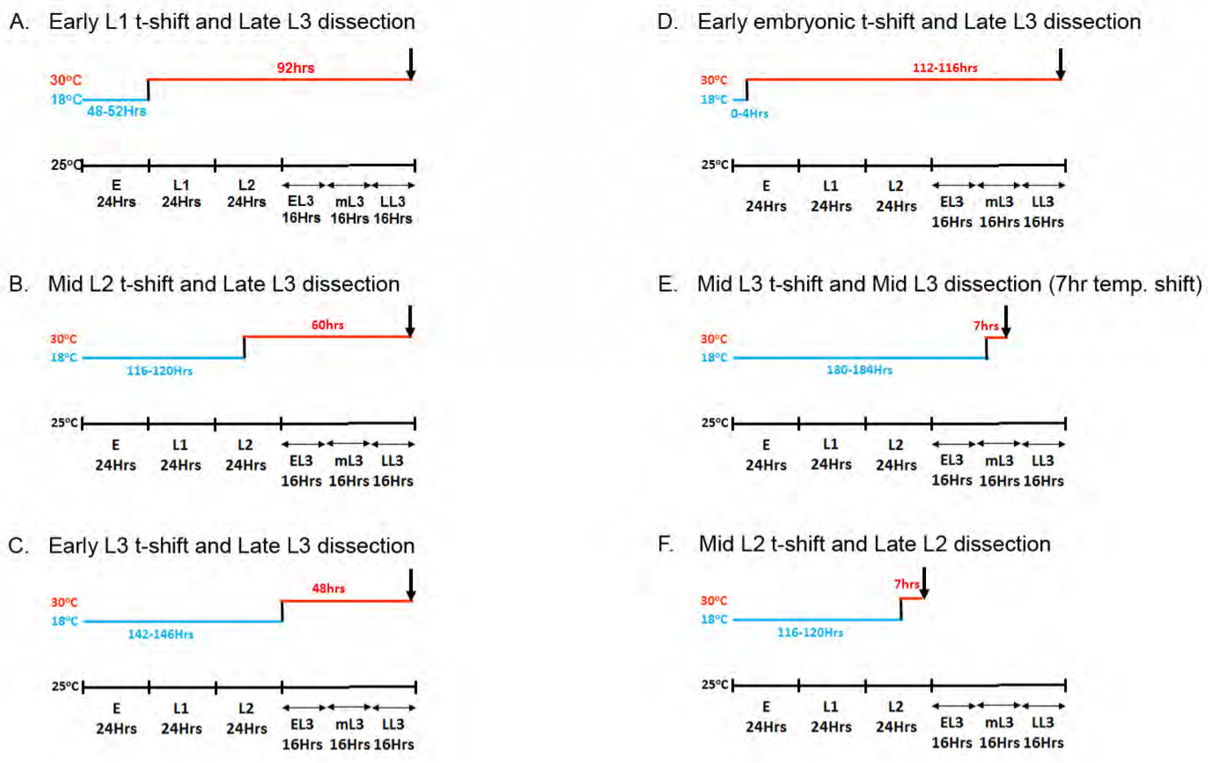

<u>Figure-S2. Approximate timings for the various temperature shift experiments</u>:

The temperature shift (TS) protocols used in different experiments in the study are shown. The downward facing arrow indicates the time of dissection of the larvae.

Development • Supplementary information

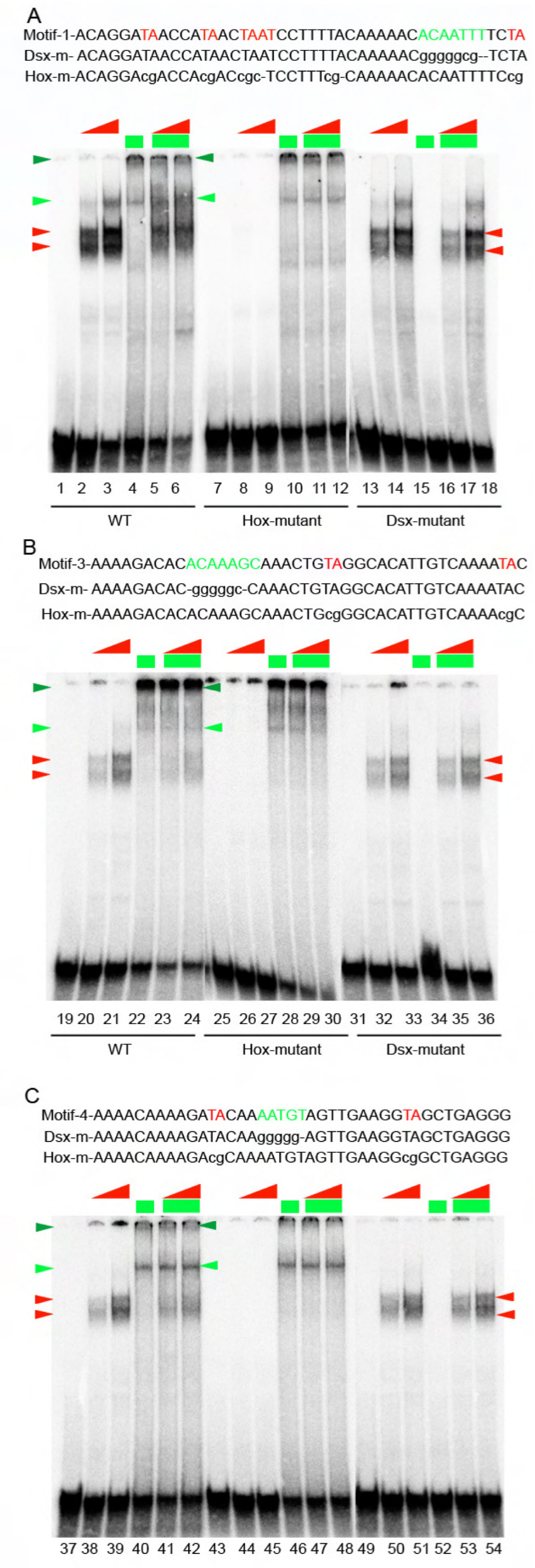

Development • Supplementary information

Figure-S3. Dsx^{CR} and AbdB does not show cooperative synergism on motif's 1, 3 and 4: EMSA for AbdB, Dsx^{CR} and AbdB-Dsx^{CR} binding on WT, Hox mutant and Dsx mutant oligos for motifs-1, 3, and 4 is shown. Hox and Dsx binding sequence are colour coded in red and green respectively. Wild type and mutant oligo sequence used are shown at the top of each gel. Mutations are shown in small case. Proteins added to a specific lane are shown at the top of each lane. Green rectangles indicate a constant concentration of 150ng for Dsx (green) protein. An increasing concentration of 50ng and 100ng of Hox are indicated by red right triangles. Dsx^{CR} used here is His-tagged. Red arrow heads on the gels indicate Hox-DNA complex; while green and dark green arrow heads indicate the Dsx monomer and dimer complex with DNA.

Development • Supplementary information

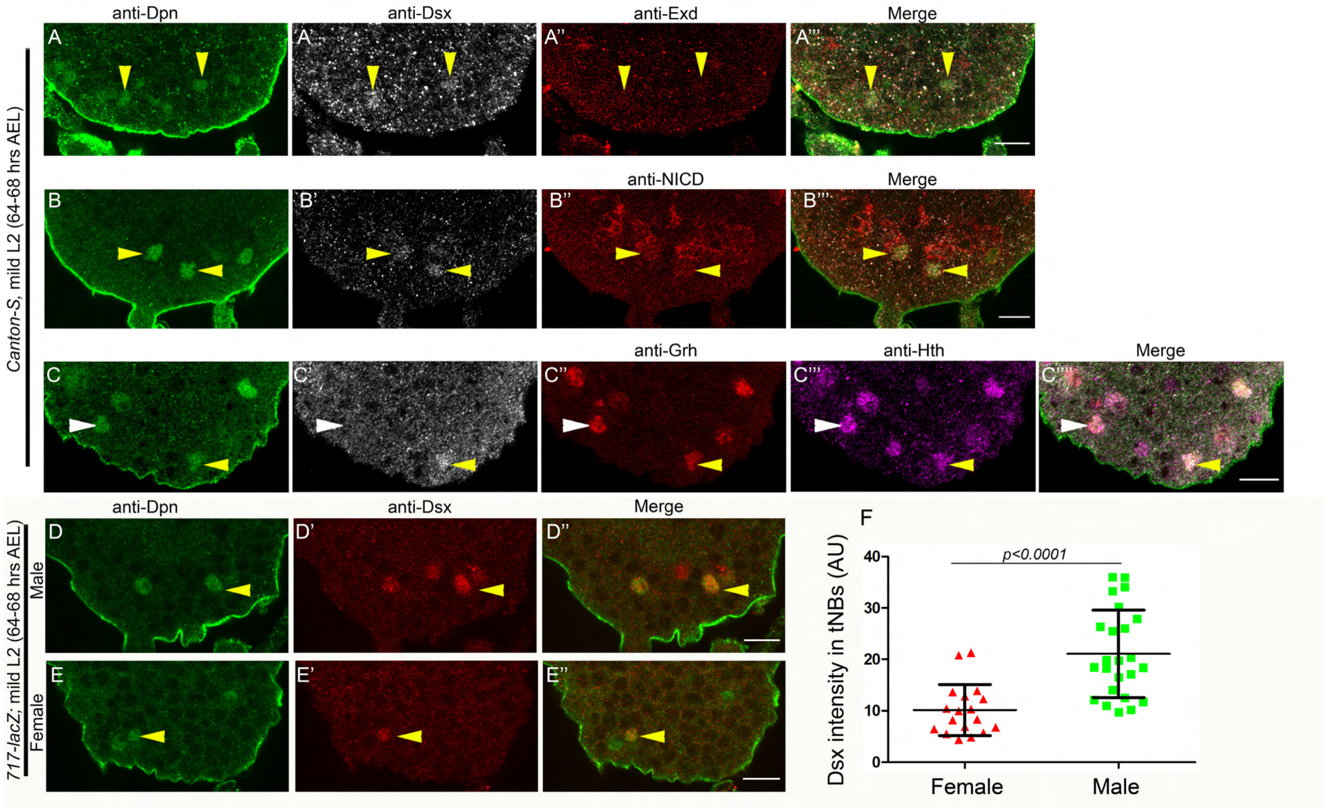

Figure-S4: Expression of Exd, Notch, Grh and Hth in Dsx^{+}tNBs in larval VNCs:

Exd (n=6) is not expressed in Dsx^{+}tNBs (A), while Notch (n=8) (B), Grh (n=10) and Hth (n=10) (C) are expressed in these cells at mid L2 stage in female VNCs. (D-E) Dsx expression levels in tNBs are sexually dimorphic, with male VNC showing higher expression compared to females. (F) Graph showing quantification of sexually dimorphic expression of levels of Dsx in tNBs in females (n=12) versus males (n=6) VNCs of *717-lacZ*. "n" indicates the number of VNCs. Significance value ("p") is from two tailed student unpaired t-test. Yellow arrowheads indicate Dsx^{+}tNBs. Scale bars is 10µm. All the images are single confocal sections. Antibodies Exd-B11M (DSHB); Notch-C17.9C6 (DSHB); Hth-GP52 (Ryoo and Mann, Development 1999), Grh (Khandelwal et al, Plos Genetics 2017); anti-Dsx rat (this study)

Development • Supplementary information

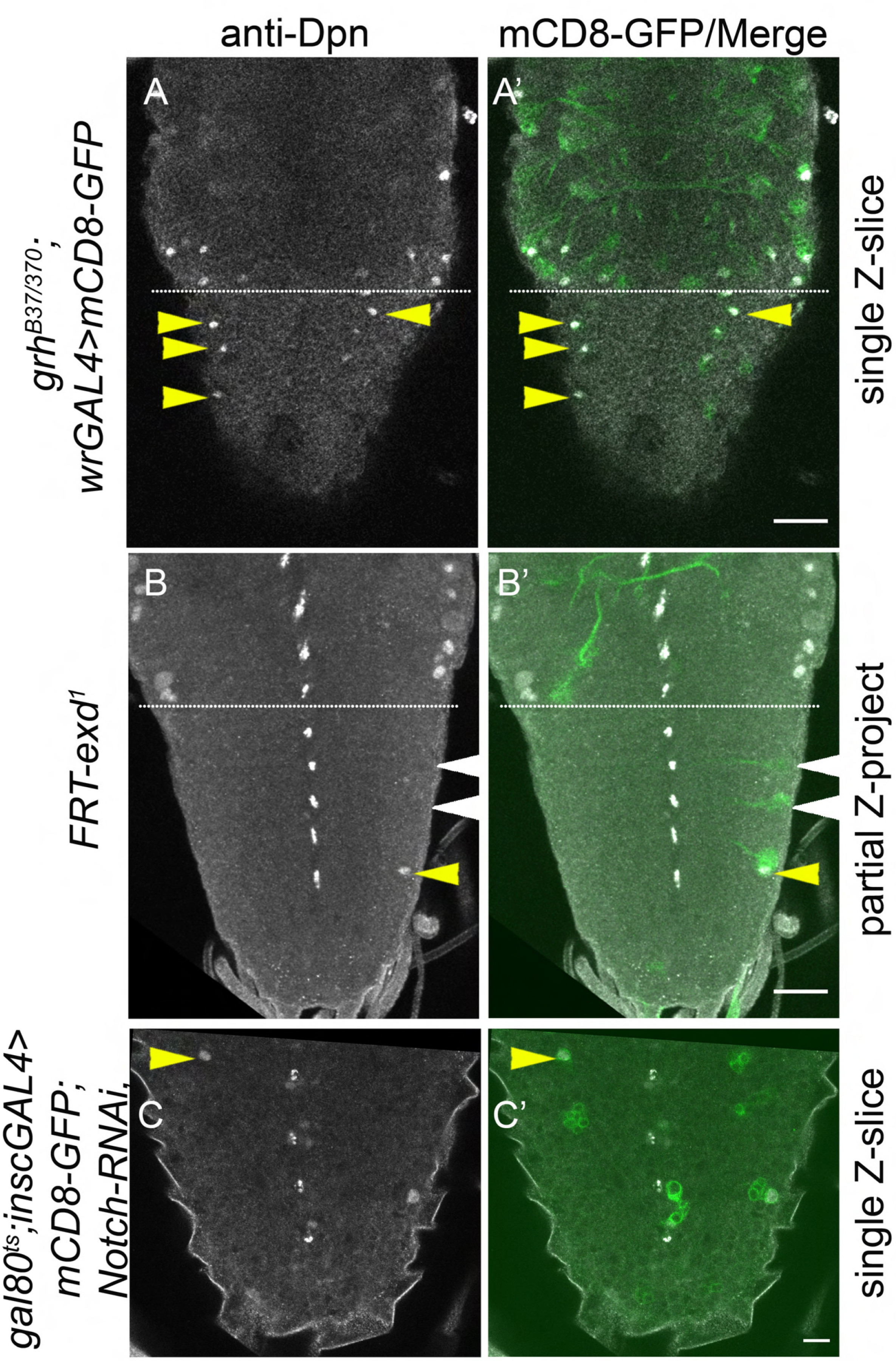

Development • Supplementary information

Figure-S5: Knockdown of Grh, Exd and Notch levels in larval VNC results in ectopic abdominal NBs: Examination of CNS specific mutant of *grh* (*grh*$^{370/B37}$) (n=15) (A), *FRT-exd*^{1} clones in abdominal segments (n'=12) (B), and knockdown of Notch by RNAi (n=16) (TS, Fig-S2A) (C), show ectopic NBs in abdominal segments of VNC, suggesting that these lines were working fine. "n" indicates the number of VNCs and "-n'-" indicates number of MARCM clones analysed. Yellow arrowheads indicate surviving NBs in abdominal segments. Scale bars is 30µm for panels-A to B and 10µm for panels-C. Z-projects and single confocal sections are indicated. Dotted line in panels-A and B show the thoracic and abdominal boundary.

Development • Supplementary information

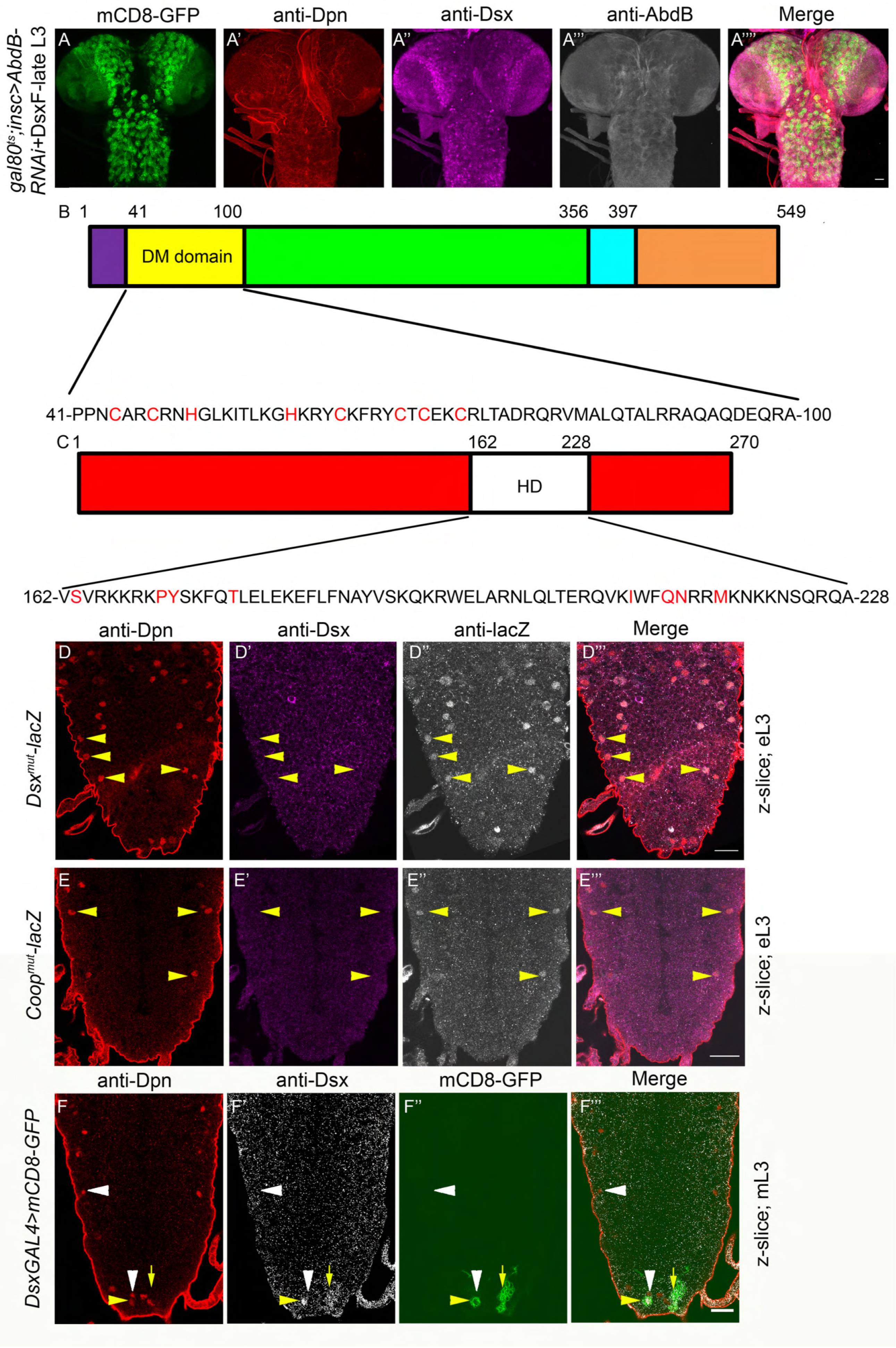

Development • Supplementary information

Figure-S6: (A-A'''') Simultaneous overexpression of Dsx^{F} and knockdown of AbdB in the female VNCs cause apoptosis in thoracic NBs in late L3 stages. Number of GFP marked thoracic NB lineages show a significant decrease (65.20+/-5.9 NBs, n=5 VNCs, N=2) (TS, Fig-S2A). Extent of DM domain of Dsx (B) and HD+6 amino acids (two N-terminal and four C-terminal) of AbdB (C) deleted for biochemical analysis is shown. Conserved residues are shown in red. (D-E) *717-Dsx*^{mutant}*-lacZ* and *717-Coop*^{mutant}*-lacZ* expresses normally in abdominal NBs in early L3 stages. This suggest that mutagenesis of Dsx binding sites or AbdB and Dsx binding sites together (in motif-2, 5 and 6) does not abrogate the enhancer activity in general. (F-F''') Anti-Dsx antibody generated in the lab, stains show specific staining in Dsx^{+}tNBs (yellow arrowheads) and in neurons of the Dsx expressing NB lineage (yellow arrows); but not in abdominal and Dsx^{-}tNBs (white arrowheads) in mid L3 stage male VNC. Yellow arrowheads in panel-D and E indicate abdominal NBs, while in panel-F they indicate Dsx^{+}tNBs. Scale bars is 20µm. panel-A is Z-projects and rest are single confocal sections as indicated.

Development • Supplementary information

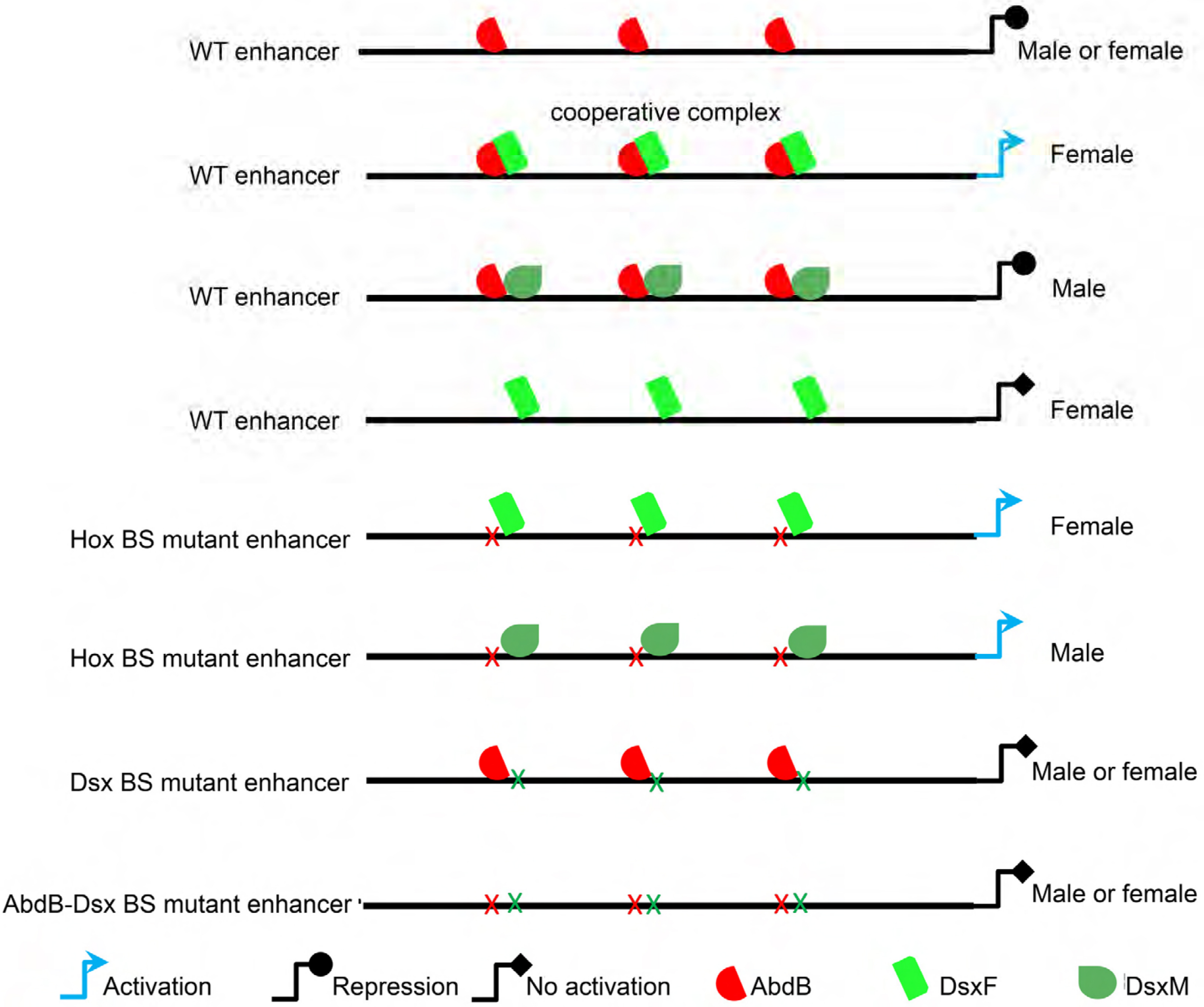

Figure-S7: Model for sex-specific apoptotic enhancer activation by AbdB and Dsx^F: Normally AbdB binds to the enhancer and keeps it repressed in both the sexes. Dsx levels increase in tNBs of males as well as females. In females AbdB and Dsx^F form a cooperative complex which activates the wild type enhancer; Dsx^F alone is not capable of activating the wild type enhancer. In males there is no cooperative complex formation and hence enhancer is kept repressed. Mutagenesis of Hox binding site prevents AbdB binding which normally act as repressor, and results in activation of the modified enhancer (in both males and females) solely by increasing levels of Dsx. Mutagenesis of Dsx binding site abrogates enhancer expression in female and shows no ectopic expression in males, suggesting that Dsx sites are critical for enhancer activation and increasing levels of Dsx^F in tNBs act as a trigger for apoptosis.

Development • Supplementary information
